# UV resonance Raman spectroscopy of the supramolecular ligand guanidiniocarbonyl indole (GCI) with 244 nm laser excitation

**DOI:** 10.3762/bjoc.16.240

**Published:** 2020-11-27

**Authors:** Tim Holtum, Vikas Kumar, Daniel Sebena, Jens Voskuhl, Sebastian Schlücker

**Affiliations:** 1 Physical Chemistry, Department of Chemistry and CENIDE, University of Duisburg-Essen, Universitätsstrasse 5, 45141 Essen, Germany; 2 Organic Chemistry, Department of Chemistry and CENIDE, University of Duisburg-Essen, Universitätsstrasse 7, 45141 Essen, Germany

**Keywords:** GCI, GCP, guanidiniocarbonyl indole, guanidiniocarbonyl pyrrole, UVRR, Raman spectroscopy, resonance Raman

## Abstract

Ultraviolet resonance Raman (UVRR) spectroscopy is a powerful vibrational spectroscopic technique for the label-free monitoring of molecular recognition of peptides or proteins with supramolecular ligands such as guanidiniocarbonyl pyrroles (GCPs). The use of UV laser excitation enables Raman binding studies of this class of supramolecular ligands at submillimolar concentrations in aqueous solution and provides a selective signal enhancement of the carboxylate binding site (CBS). A current limitation for the extension of this promising UVRR approach from peptides to proteins as binding partners for GCPs is the UV-excited autofluorescence from aromatic amino acids observed for laser excitation wavelengths >260 nm. These excitation wavelengths are in the electronic resonance with the GCP for achieving both a signal enhancement and the selectivity for monitoring the CBS, but the resulting UVRR spectrum overlaps with the UV-excited autofluorescence from the aromatic binding partners. This necessitates the use of a laser excitation <260 nm for spectrally separating the UVRR spectrum of the supramolecular ligand from the UV-excited autofluorescence of the peptide or protein. Here, we demonstrate the use of UVRR spectroscopy with 244 nm laser excitation for the characterization of GCP as well as guanidiniocarbonyl indole (GCI), a next generation supramolecular ligand for the recognition of carboxylates. For demonstrating the feasibility of the UVRR binding studies without an interference from the disturbing UV-excited autofluorescence, benzoic acid (BA) was chosen as an aromatic binding partner for GCI. We also present the UVRR results from the binding of GCI to the ubiquitous RGD sequence (arginylglycylaspartic acid) as a biologically relevant peptide. In the case of RGD, the more pronounced differences between the UVRR spectra of the free and complexed GCI (1:1 mixture) clearly indicate a stronger binding of GCI to RGD compared with BA. A tentative assignment of the experimentally observed changes upon molecular recognition is based on the results from density functional theory (DFT) calculations.

## Introduction

**Figure 1:**
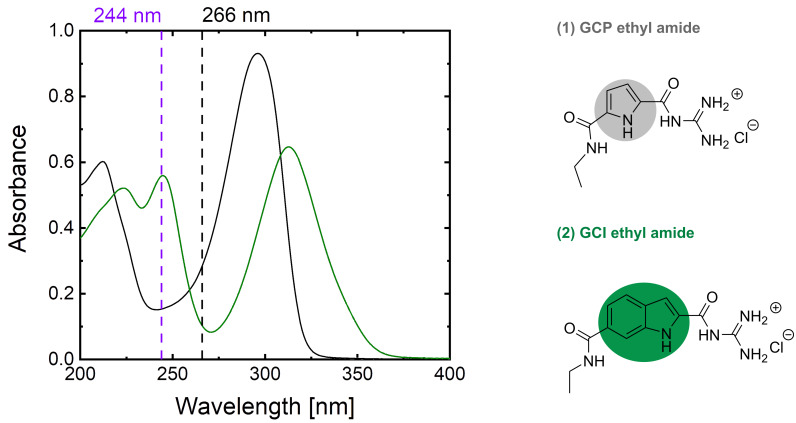
UV–vis absorption spectra of GCP ethyl amide [9] (in grey) and GCI ethyl amide (in green) at 200 µM concentration in 6 mM BisTris buffer solution at pH 6. The excitation wavelengths used for the UVRR experiments are indicated as dashed lines. The schematic molecular structures of the protonated GCP ethyl amide and protonated GCI ethyl amide are also displayed. The substructures highlighted in grey and green are pyrrole and indole rings, respectively.

Supramolecular ligands are capable to selectively bind to peptides and proteins via reversible non-covalent interactions namely hydrogen bonds, van der Waals, and/or hydrophobic interactions [1–5]. In this context, Schmuck and co-workers have introduced a class of synthetic receptors based on the guanidiniocarbonyl pyrrole (GCP) moiety (cf. Figure 1 top right) as a carboxylate binding site (CBS) [6–9]. The GCP takes part selectively and efficiently in the complexation of carboxylates based on the electrostatic interaction between the positively charged CBS and the negatively charged carboxylate in the combination with hydrogen bonding, enabling molecular recognition even in the presence of polar solvents like water. This makes GCPs promising binding partners for acidic residues such as carboxylates at the C-terminus of peptides and proteins.

The intermolecular interactions upon recognition induce subtle changes in the molecular properties such as the electronic structure and bond strengths. Various spectroscopic techniques can be employed for monitoring these changes. For example, electronic absorption or fluorescence spectroscopy can probe the spectral differences due to the complexation of the supramolecular ligand with a peptide or protein. However, electronic spectroscopies probe the entire chromophore and are sensitive only to changes in the electronic structure of the molecule. In contrast, vibrational spectroscopic techniques such as infrared (IR) and Raman spectroscopy provide a much more detailed picture at the level of chemical bonds since they probe the intrinsic vibrational modes of the molecule. Especially for non-covalent interactions such as hydrogen bonding, vibrational spectroscopy has been shown to be very sensitive [10–11]. In the context of supramolecular recognition, for example, IR spectroscopy has been applied to monitor the binding of tetrapeptides by GCP-based supramolecular ligands containing also a tripeptide part for increasing selectivity [12]. However, IR spectroscopy does not provide selectivity for probing only the CBS. This is not critical as long as the binding partner is a small peptide, i.e., when the spectrum is not too crowded because of the small number of vibrational peaks. Additionally, IR spectroscopy suffers from the strong absorption by water. In contrast, this is not a problem in Raman spectroscopy because water is a weak Raman scatterer. Conventional Raman spectroscopy under nonresonant conditions is typically limited to millimolar concentrations due to the small Raman scattering cross sections. Therefore, the biologically relevant submillimolar concentrations of GCP are not detectable by conventional (off-resonant) Raman spectroscopy. This limitation can be overcome by resonance Raman (RR) spectroscopy because it offers a very high sensitivity owing to its enhanced Raman scattering from molecules that are in electronic resonance with the excitation laser frequency. In case of supramolecular recognition, this gives the additional advantage of selectivity where a molecular subunit can be selectively excited by properly choosing the laser wavelength in electronic resonance and the enhanced Raman spectrum involving only vibrational modes of the molecular subunit is selectively detectable. Since the GCP exhibits electronic resonances in the ultraviolet region with an absorption maximum at ca. 298 nm (see UV–vis absorption spectrum of GCP in Figure 1, left), ultraviolet resonance Raman (UVRR) spectroscopy is employed for selectively enhancing the Raman signal from the GCP subunit. UVRR spectroscopy gives signal enhancements by several orders of magnitude, enabling UVRR binding studies of GCP at submillimolar concentrations. In earlier studies, we have demonstrated the suitability of UVRR spectroscopy for monitoring the supramolecular binding of monovalent GCP-based ligands with peptides [12–16] and a trivalent GCP-based ligand with the protein leucine zipper, a protein with a single aromatic unit [17], by using 275 and 266 nm laser excitation, respectively.

**Figure 2:**
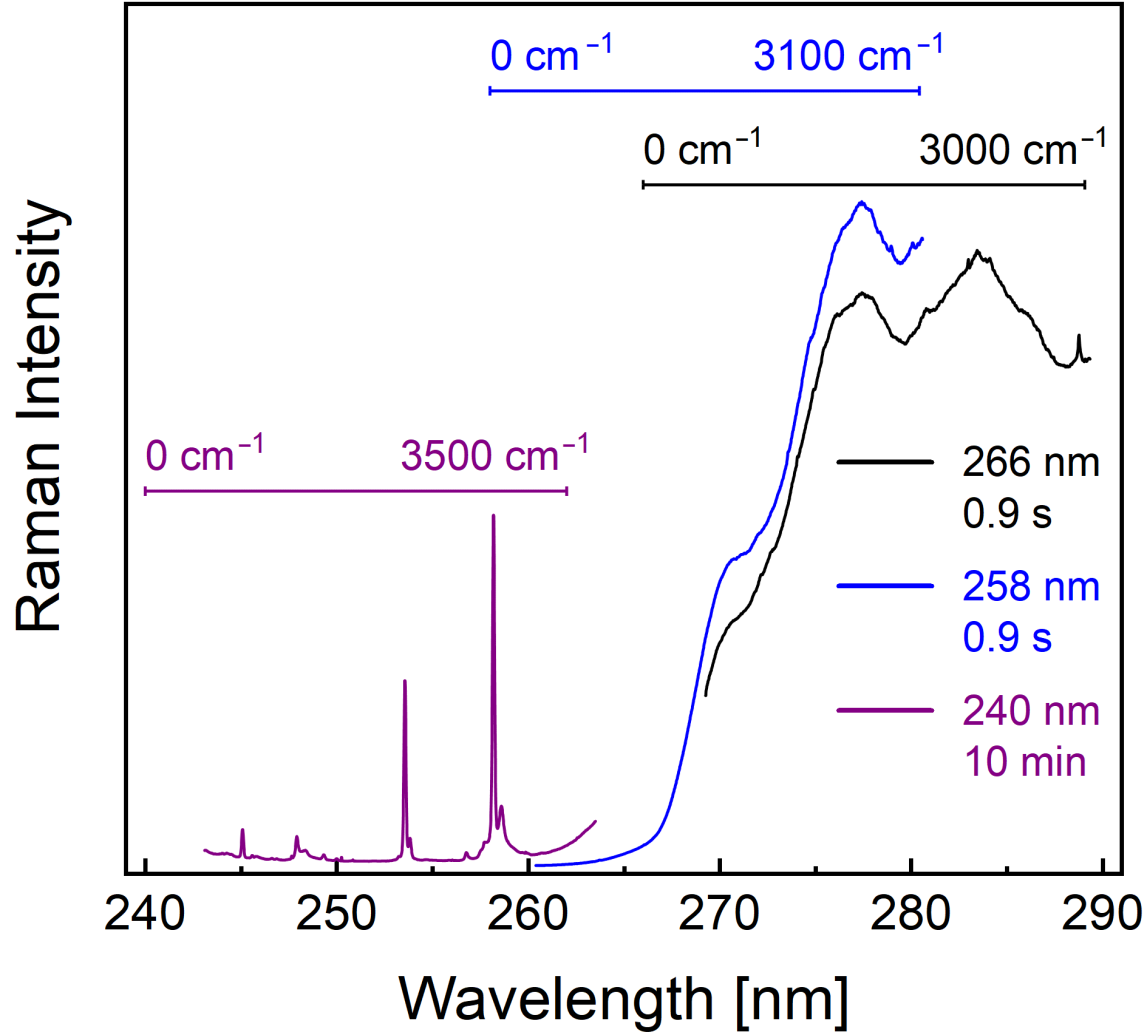
UVRR spectra of a 1 mM solution of toluene in acetonitrile acquired with 240, 258, and 266 nm laser excitation, respectively.

The current challenge with UVRR spectroscopy for monitoring the recognition of supramolecular ligands to proteins is the disturbing UV-excited autofluorescence from the aromatic amino acids. This autofluorescence typically occurs in the spectral range 260–440 nm and can significantly mask the spectrally overlapping UVRR signal. Due to this reason, UVRR binding studies so far were limited to the proteins with no or a minimal number of aromatic residues, for example, a leucine zipper with one phenylalanine [17]. However, the problem can be circumvented either by temporally discriminating the Raman signal from the autofluorescence by using an optical switch such as a Kerr gate [18–19], or by using short excitation wavelengths for spectrally separating the UVRR signals from the UV-excited autofluorescence. The latter approach is achieved by sufficiently blue-shifting the UVRR spectrum away from the UV-excited autofluorescence [20–21]. This is illustrated in Figure 2 for the UVRR spectra of a 1 mM solution of toluene in acetonitrile acquired with 240, 258, and 266 nm laser excitation. The spectra are plotted as a function of the wavelength (rather than the relative wavenumber/Raman shift) to illustrate the blue-shifting of the UVRR spectral range of interest (0–3000 cm^−1^) when moving towards the lower excitation wavelengths. It can be seen that the spectral position of the UV-excited autofluorescence is independent of the choice of the excitation wavelength and that it overwhelms the detector even at a short integration time (0.9 s) for 258 and 266 nm excitations, masking the spectrally overlapped much weaker Raman signals. On the other hand, the 240 nm laser excitation spectrally isolates the UVRR spectral from the UV-excited autofluorescence. This allowed us to use a sufficiently high integration time (10 minutes) for obtaining good quality UVRR spectra at 244 nm excitation.

In this study, we evaluate the performance of UVRR spectroscopy with 244 nm laser excitation for the characterization of two guanidiniocarbonyl-based supramolecular ligands: guanidiniocarbonyl pyrrole (GCP) and guanidiniocarbonyl indole (GCI). The latter class of artificial carboxylate receptors is a potential next generation binder based on the GCI motif which maintains the good carboxylate binding properties of GCP. GCI comprises an indole ring instead of a pyrrole ring, which leads to different optical absorption properties in the UV–visible range (see Figure 1). Based on the UV–vis absorption spectrum, we expect a stronger resonance enhancement for GCI than GCP at 244 nm excitation due to its higher absorbance. Again, a 244 nm excitation is necessary for spectrally separating the UVRR signal (below 260 nm) from the UV-excited autofluorescence starting from ca. 260 nm (cf. Figure 2) [22]. We employ two different carboxylates for the UVRR binding studies: benzoic acid (BA) as an aromatic model system and the ubiquitous RGD sequence (arginylglycylaspartic acid). BA was chosen in order to test experimentally whether the UVRR at 244 nm laser excitation is free from UV-excited autofluorescence as expected from the initial results with toluene (Figure 2). RGD was chosen as a biologically relevant tripeptide sequence highly abundant in proteins of the extracellular matrix.

## Results and Discussion

**Figure 3:**
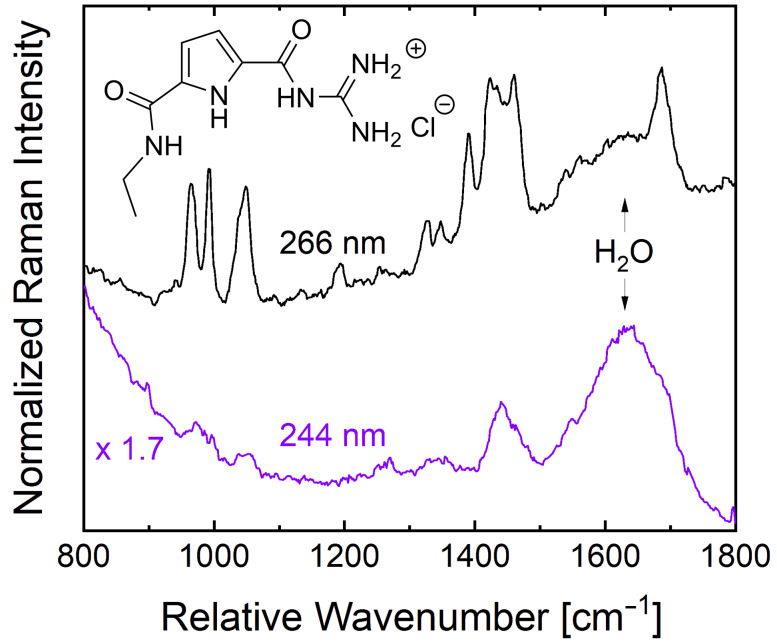
The UVRR spectra of a 200 µM solution of GCP ethyl amide in 6 mM BisTris buffer solution at pH 6 acquired with 266 nm (black curve) and 244 nm (magenta curve) laser excitation, respectively.

Figure 1 (left) displays the absorption spectra of GCP ethyl amide and GCI ethyl amide at 200 µM concentration in buffer solution. The GCP ethyl amide [9] shows a strong electronic absorption covering the entire spectral region below ca. 325 nm with two peaks at 298 and 215 nm. Both absorption bands can be attributed to π→π* transitions within the GCP chromophore. On the other hand, GCI ethyl amide exhibits a slightly red-shifted absorption band covering the entire region below 370 nm with two peaks at 320 and 244 nm which are also assigned to π→π* transitions of the GCI chromophore. The red-shift in the spectral positions of the GCI peaks with respect to those of the GCP peaks is due to the extended conjugation induced by the indole ring of GCI. We chose 266 nm and 244 nm as laser excitation wavelengths for UVRR spectroscopy. The 266 nm excitation was chosen because it has been employed in a recent UVRR binding study of a trivalent GCP-based ligand with the protein leucine zipper. The 244 nm excitation was chosen because this wavelength is sufficiently below the 260 nm minimum mark for avoiding spectral interference of the UVRR with UV-excited autofluorescence and because it matches an intense electronic absorption peak of GCI. First, we performed UVRR spectroscopy on a 200 µM GCP solution using 266 and 244 nm laser excitation (Figure 3). The UVRR spectrum with 266 nm excitation (black curve) shows the characteristic strong Raman bands in the region 800–1800 cm^−1^. Peaks appearing within the 900–1100 cm^−1^ region cover various pyrrole ring deformation modes while the peak at 1400 cm^−1^ belongs to a symmetrical half-ring vibration of pyrrole [13]. Based on the results from DFT calculations [13], the peaks around 1470 cm^−1^ are assigned to N–H bending and C–N stretch modes of both guanidinio and pyrrole. The peak at 1697 cm^−1^ has a contribution from an Amide I-like vibration with C=O stretch contributions at the guanidiniocarbonyl part of the receptor.

In contrast, at 244 nm laser excitation only few, broad, and featureless Raman peaks are observed (violet curve). This weaker resonance enhancement is due to the weaker electronic absorption of the GCP chromophore at 244 nm (see Figure 1). The dominant and broad Raman band around 1640 cm^−1^ is the deformation mode of water. This water band can also be seen in the 266 nm-excited UVRR spectrum. Overall, due to the low signal enhancement in the UVRR spectrum obtained with 244 nm laser excitation, we did not perform any UVRR binding studies for GCP at the wavelength. Again, this shorter excitation wavelength compared to 266 nm is necessary for circumventing the UV-autofluorescence occurring from aromatic binding partners.

**Figure 4:**
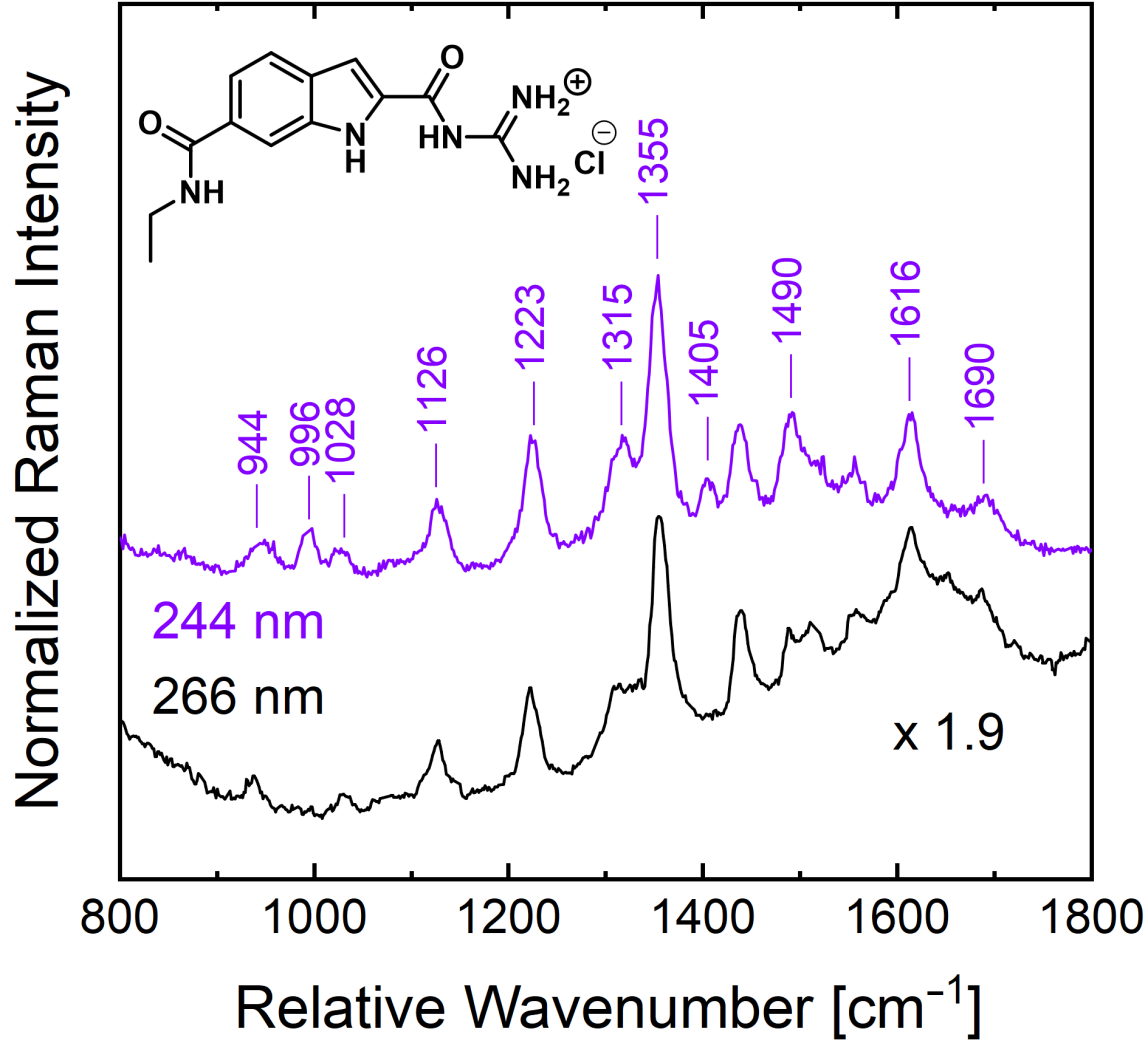
The UVRR spectra of GCI ethyl amide at a 200 µM concentration in 6 mM BisTris buffer solution at pH 6 acquired with 266 nm (black curve) and 244 nm (magenta curve) laser excitations, respectively.

We therefore performed a UVRR characterization of GCI at 266 nm and 244 nm laser excitation. From the strong electronic absorption of GCI (Figure 1) a strong enhancement of the UVRR signal from GCI is expected for the 244 nm excitation. Figure 4 shows the UVRR spectra obtained from a 200 µM GCI solution using 266 and 244 nm laser excitation, respectively. In contrast to the spectra for the GCP ethyl amide, both UVRR spectra of GCI ethyl amide show many sharp Raman bands. The intensities in the GCI UVRR spectrum with 244 nm laser excitation are twice as high compared to those in the UVRR spectrum recorded with the 266 nm excitation. The strong signal for 244 nm excitation is explained by the higher absorbance of GCI at 244 nm compared to the absorbance at 266 nm. Both GCI UVRR spectra look very similar, exhibiting several distinct bands with the strongest peak around 1356 cm^−1^. Upon moving the laser excitation from 266 nm to 244 nm, two peaks around 996 cm^−1^ and 1405 cm^−1^ are additionally enhanced and can be detected. Further, we observe a relative intensity increase for the band at 1490 cm^−1^.

For a peak assignment of the experimental UVRR spectrum of GCI, we employed the results from density functional theory (DFT) calculations. The assignment of the peaks detectable in the experimental UVRR spectrum is mainly based on the experimental wavenumber position (peak position). Only normal modes with non-vanishing Raman activities were considered. One cannot expect an agreement between the experimental and theoretical intensities since the DFT calculations do not include the electronic resonance enhancement, which was exploited in the UVRR experiments.

**Figure 5:**
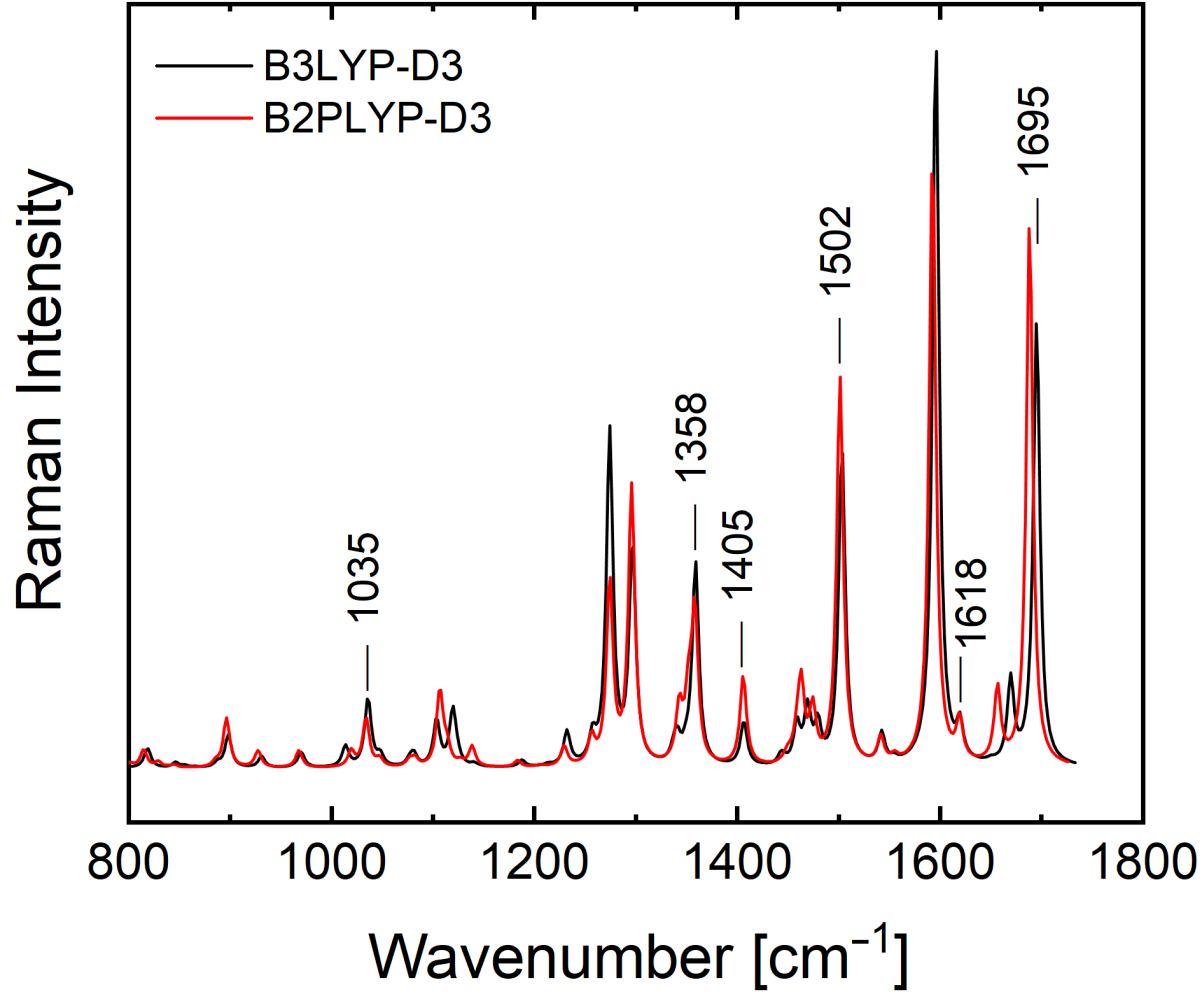
The computed Raman spectrum of GCI ethyl amide in the fingerprint region 800–1800 cm^−1^, calculated at the B3LYP-D3/6-311++G(d,p) and B2PLYP-D3/6-311++G(d,p) level of theory.

Figure 5 presents the theoretical Raman spectra of the GCI ethyl amide molecule in the region 800–1800 cm^−1^ calculated at the B3LYP-D3/6-311++G(d,p) and the B2PLYP-D3/6-311++G(d,p) level of theory. A complete list of the calculated vibrational modes along with their wavenumber positions and corresponding Raman activities is available in Supporting Information File 1. Both calculated spectra show similar results. For our discussion, we evaluate the data of the calculations from the more common B3LYP-D3 functional. Overall, there is a good agreement between the positions of the eleven peaks labeled in the UVRR spectra in Figure 4 and the calculated wavenumbers after multiplication with a global scaling factor: 944 cm^−1^ (theo. 930 cm^−1^), 996 cm^−1^ (theo. 1011 cm^−1^), 1028 cm^−1^ (theo. 1035 cm^−1^), 1126 cm^−1^ (theo. 1115 cm^−1^), 1223 cm^−1^ (theo. 1227 cm^−1^), 1315 cm^−1^ (theo. 1335 cm^−1^), 1355 cm^−1^ (theo. 1358 cm^−1^), 1405 cm^−1^ (theo. 1405 cm^−1^), 1490 cm^−1^ (theo. 1502 cm^−1^), 1615 cm^−1^ (theo. 1618 cm^−1^) and 1690 cm^−1^ (theo. 1695 cm^−1^).

However, as expected, the relative intensities of the modes in the experimental UVRR spectra cannot be properly predicted by theory which calculated the nonresonant Raman intensities. Calculating UVRR spectra for a complex molecule such as GCI is very challenging and beyond the scope of this paper.

**Figure 6:**
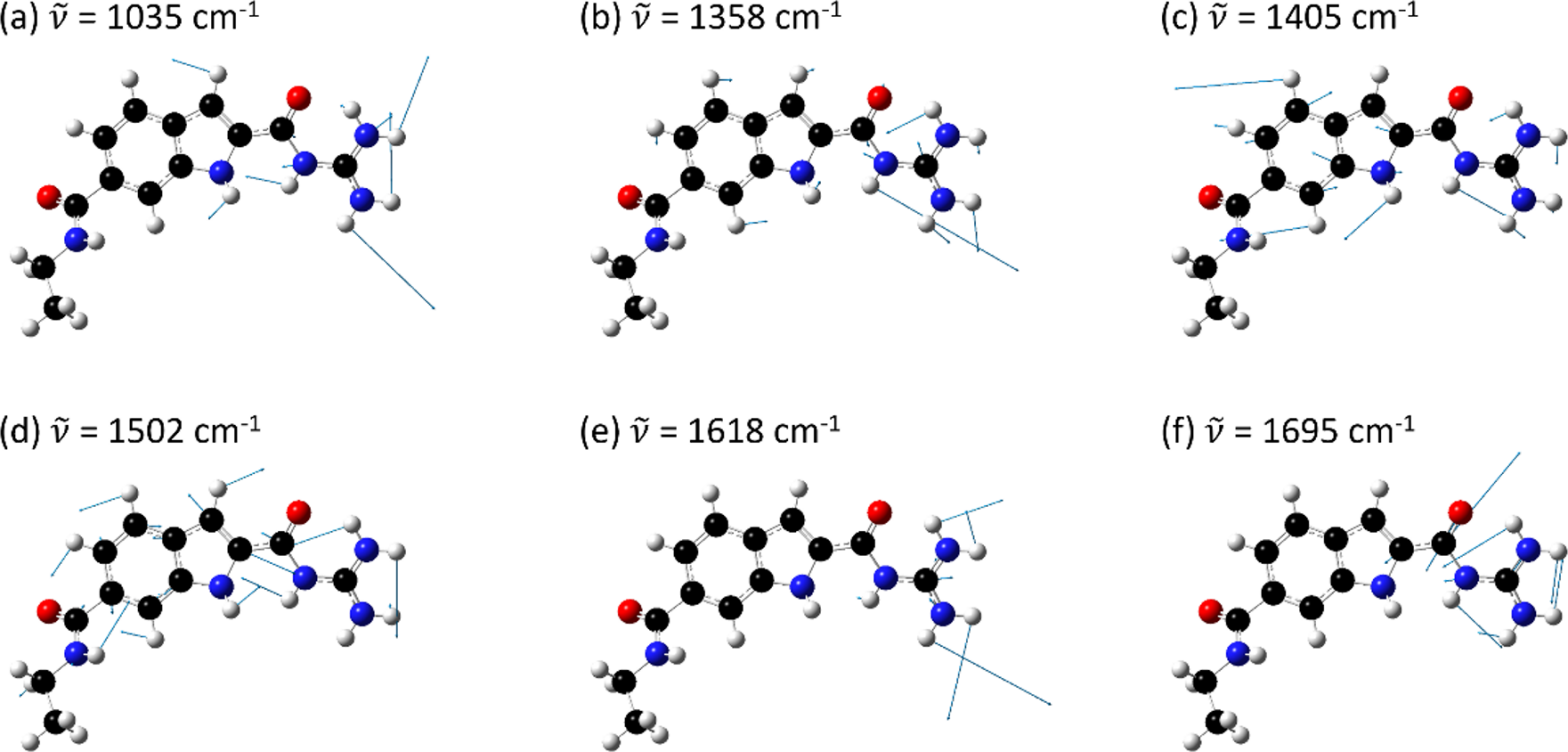
The DFT computed eigenvectors of GCI ethyl amide of selected normal modes (cf. Figure 5) at (a) 1035 cm^−1^, (b) 1358 cm^−1^, (c) 1405 cm^−1^, (d) 1502 cm^−1^, (e) 1618 cm^−1^, and (f) 1695 cm^−1^.

Figure 6 shows the DFT-calculated eigenvectors of six selected GCI modes at 1035 cm^−1^, 1358 cm^−1^, 1405 cm^−1^, 1502 cm^−1^, 1618 cm^−1^, and 1695 cm^−1^ which show a significant involvement of the guanidinio group. The 1035 cm^−1^ peak has a contribution from a rocking motion of the guanidinio group extending to the pyrrole part of the indole ring (symmetric C–N stretching and asymmetric N–H bending), while the peak at 1358 cm^−1^ belongs to vibrations mainly located at the guanidinio group C–N stretch and N–H bending (amide III like). The 1405 cm^−1^ peak can be attributed mainly to a stretching mode of the indole ring (similar to fundamental mode ν_12_ of indole in ref. [23]), and additionally to the N–H bending at the guanidinio group. The peak at 1502 cm^−1^ has a major contribution from the dominant in-plane ring stretching mode of the indole (ν_10_ in ref. [23]) and N–H bendings from all N–H groups (peptide, indole, guanidinio) as well as a minor contribution from a C–N stretch. The mode at 1616 cm^−1^ consists of C–N stretching and N–H bending located at the guanidinio group, while the one at 1695 cm^−1^ belongs to the amide I-like vibration involving a C=O stretch at the guanidiniocarbonyl part of the receptor. The vibrational assignment of these modes is important for the binding study of GCI receptors because we hypothesize that they are all involved in the complexation with carboxylates.

Finally, we employed UVRR spectroscopy at 244 nm excitation to observe the binding events of GCI-based receptors using two different carboxylates as the binding partners. We choose an aromatic binding partner, benzoic acid, for which the UV-excited autofluorescence might disturb the Raman signal at higher excitation wavelengths. For the second binding experiment we choose a biologically relevant peptide, RGD.

**Figure 7:**
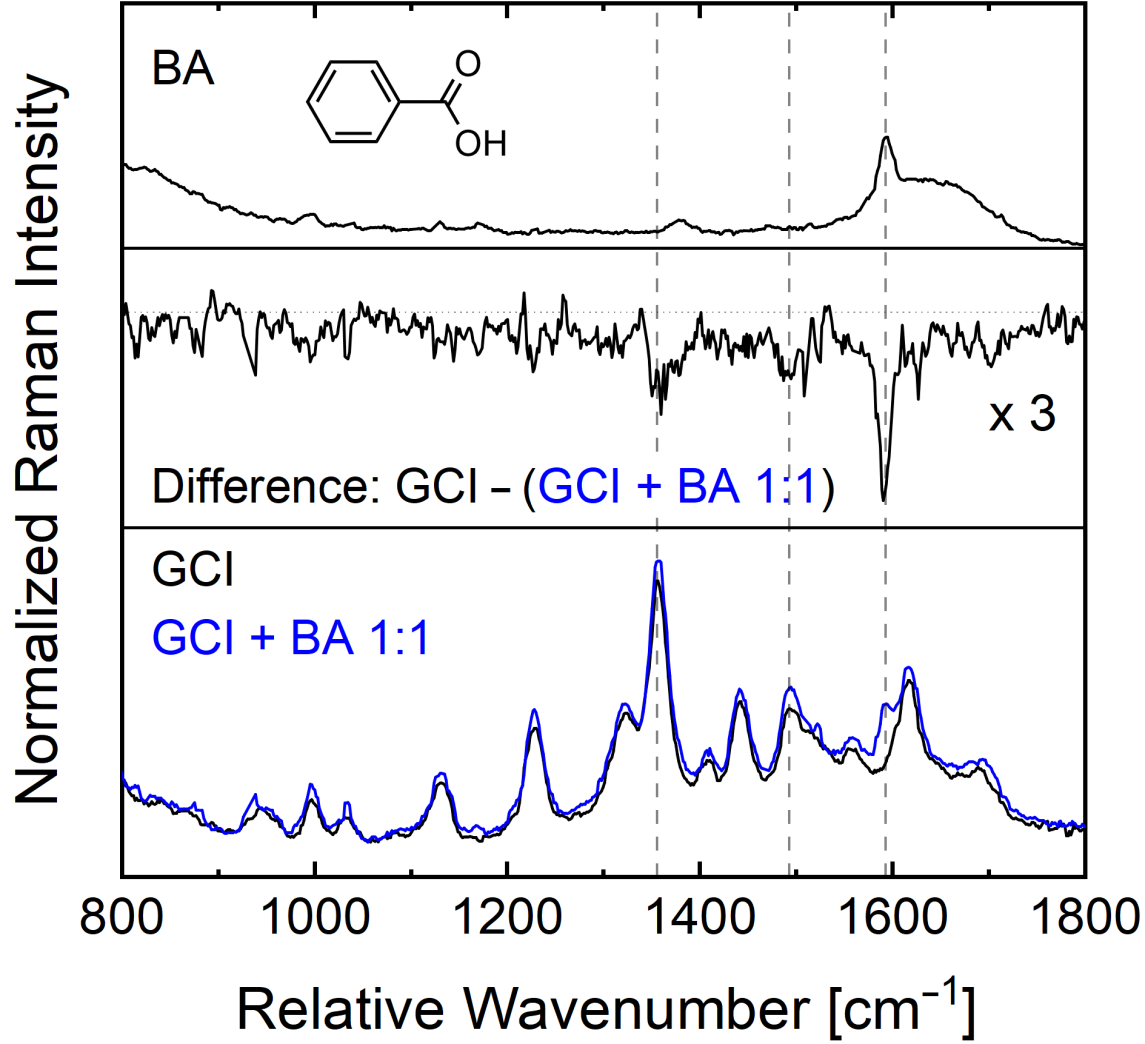
The UVRR spectra obtained with 244 nm laser excitation: GCI and a 1:1 mixture of ‘GCI and BA’ (bottom), the difference spectrum (middle), and BA (top). The dashed lines mark the most prominent changes in the difference spectrum.

In the first experiment, the UVRR spectrum of a 1:1 mixture of benzoic acid and GCI ethyl amide (both 200 µM) was acquired with 244 nm laser excitation (shown as blue curve in Figure 7). This spectrum is compared to the UVRR spectra of the corresponding binding partners GCI ethyl amide (200 µM, black curve in Figure 7 bottom) and of BA (200 µM, Figure 7 top) both acquired maintaining the same experimental conditions. All three spectra were normalized to the Raman peak of water (OH stretching) around 3400 cm^−1^. The UVRR spectra of the GCI + BA mixture and GCI show similar features with slight variations in the intensity levels. To highlight the changes between the two spectra, the difference spectrum is plotted in Figure 7 (middle). Prominent changes can be observed at 1596 cm^−1^, 1490 cm^−1^, and 1355 cm^−1^. The band at 1596 cm^−1^ can be assigned to BA. However, the band at 1355 cm^−1^ which belongs to vibrations of the guanidinio group C–N stretch and N–H bending, and the band at 1501 cm^−1^ which has the major contribution from the dominant ring-stretching mode of indole, are expected to take part in the carboxylate binding. The observation of intensity variations for these bands indicates the carboxylate binding of GCI and BA. However, such subtle changes suggest that BA is a weak binding partner for GCI for molecular recognition.

**Figure 8:**
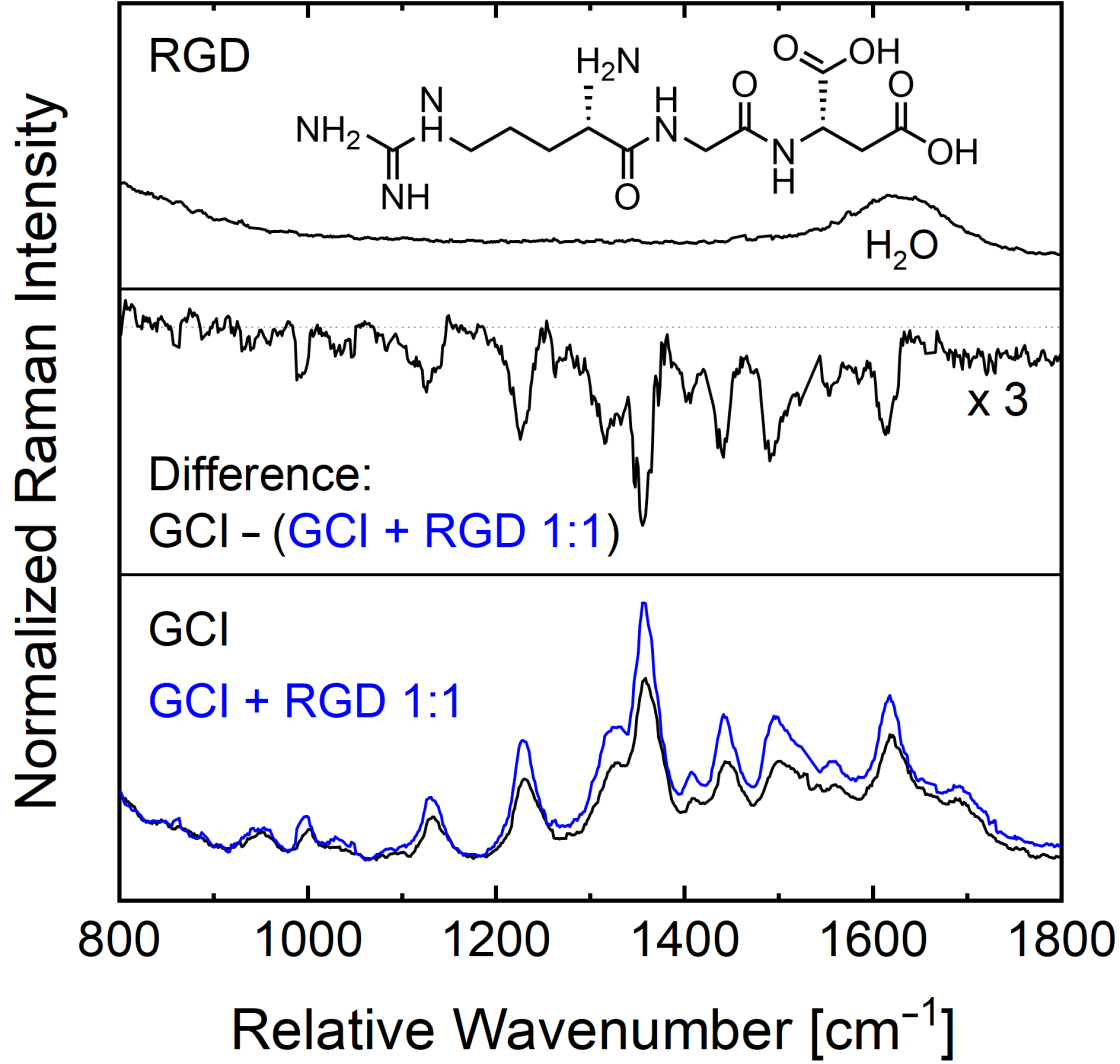
The UVRR spectra obtained with 244 nm laser excitation: GCI and a 1:1 mixture of ‘GCI and RGD’ (bottom), difference spectrum (middle), and RGD (top).

In a second binding experiment, we used the biologically relevant tripeptide RGD as a binding partner. The UVRR spectrum of a 1:1 mixture of RGD and GCI ethyl amide (both 200 µM) was acquired with 244 nm laser excitation (Figure 8, blue curve). For comparison, the UVRR reference spectra of the two isolated components, i.e., 200 µM GCI (Figure 8, bottom black curve) and 200 µM RGD (Figure 8, top), were also recorded maintaining the same experimental conditions. All three spectra were then normalized to the Raman peak of water (OH stretching) at ca. 3400 cm^−1^. A comparison of the UVRR spectrum of the 1:1 mixture of GCI and RGD with the GCI reference spectrum shows strong intensity variations across a wide spectral range. Overall, these intensity changes are much more pronounced compared to the binding experiment with BA, where intensity variations just for few particular bands were observed (Figure 7).

In the following paragraph, we provide an explanation on why, upon complexation no peak shifts but only intensity differences are observed. Even though there are only non-covalent, i.e., only weak intermolecular interactions present, one would expect a peak shift for the isolated supramolecular ligand. However, the GCI is dissolved in water and therefore already involved in hydrogen bonding to water molecules. Upon the addition of the RGD binding partner, the water molecules in the binding pocket are “replaced”. This is why, in summary, we only observe intensity changes upon the complexation of GCI (this work) and GCP (earlier published work). There are several possible reasons for an increased Raman intensity upon complexations: an increased static polarizability due to conformational changes and/or increased transition polarizabilities due to minor changes of the ground and excited state potential.

The difference spectrum (Figure 8, middle) shows strong peaks at 1033 cm^−1^, 1120 cm^−1^, 1225 cm^−1^, 1310 cm^−1^, 1355 cm^−1^, 1405 cm^−1^, 1450 cm^−1^, 1501 cm^−1^, 1616 cm^−1^, and 1695 cm^−1^. Thus, the corresponding normal modes of these peaks are involved in the complexation of the GCI. For six of these peaks the calculated eigenvectors in Figure 6 clearly show an involvement of vibrations with contributions located at the indole ring and/or the guanidinio moiety. Overall, the more pronounced changes in terms of larger intensities in the difference spectrum also suggest that RGD is a stronger binding partner to GCI as compared to BA. This is expected due to the fact that RGD has two carboxylic groups per molecule as compared to only one in BA (cf. the molecular structures of BA and RGD in Figure 7 and Figure 8, respectively).

## Conclusion

Applying UVRR for label-free monitoring of molecular recognition of proteins by supramolecular ligands requires that the UV-induced autofluorescence is circumnavigated. This can be realized either in the time domain by using a UV Kerr-gate for temporally separating the Raman scattering from the time-delayed fluorescence or in the frequency domain by using shorter excitation wavelengths for blue-shifting the UVRR signal away from the UV-excited autofluorescence. In this study, we have demonstrated the performance of UVRR spectroscopy with 244 nm and 266 nm laser excitation for two supramolecular ligands: GCP and GCI as a next generation binder. We have shown that for GCI the second option in the frequency domain works since the resonance enhancement is sufficient. However, for GCP this does not work due to the insufficient resonance enhancement. We also observed GCI binding events with two distinct binding partners: BA and RGD. RGD is found to be a stronger binding partner for GCI. We employed the results from DFT for an assignment of the peaks in the experimental UVRR spectrum of GCI. For future UVRR binding studies with proteins using the GCP motif, the development of a Kerr-gate operating in the UV below 300 nm is required.

## Experimental

For the 244 nm laser excitation a pulsed laser source (Light Conversion; Pharos, Orpheus-PS, SHBC, LYRA) providing pulses at 10 kHz repetition rate with the wavelength continuously tunable in the 210–350 nm UV region was employed. For the 266 nm laser excitation a continuous wave (CW) laser source (CryLaS GmbH, FQCW 266) was used. The UVRR spectrometer comprises a 50 cm focal length grating monochromator (Acton Research Corp., SpectraPro-500i, 2400 grooves/mm grating) equipped with a cryogenically-cooled CCD sensor (Princeton Instruments, PyLoN:2KBUV). To avoid the possible interference by sample container material and to eliminate sample degradation by excess exposure to UV light, the liquid sample was circulated in a home-built free-flow system similar to the one employed for deep UVRR studies [24]. This closed-loop system is driven by a peristaltic pump. A half air-filled syringe acts as an upper reservoir to flatten pressure oscillations from the peristaltic pump. An injection needle with an 0.8 mm outer diameter is attached to the syringe as the output nozzle to form a laminar liquid column in air at the laser focus. The laser radiation was focused by two cylindrical lenses to create a line focus along the sample liquid column and a 90° scattering geometry was used for collecting the Raman scattered light.

The UV–vis absorption spectra were acquired with a UV–vis spectrometer (Perkin-Elmer Lambda 650) where liquid samples were kept in 2 mm fused silica cuvettes (Hellma). DFT calculations were performed with the Gaussian 16 program package [25]. The molecule was calculated in the gas phase in its protonated form with one positive net charge. The resulting wavenumber values were scaled by a factor of 0.964 [26] and 0.960 for B3LYP-D3 and B2PLYP-D3, respectively. A HWHM of 4 cm^−1^ and a Lorentzian line shape were used for simulating the theoretical spectrum in Figure 5. The GCP building block was synthesized according to a known literature procedure [27], followed by further functionalization with ethylamine at the carboxylic acid in two steps to obtain the GCP ethyl amide, which has been synthesized before, by a different method [9]. The novel building block for GCI was synthesized in a 4-step synthesis adapted from a previous work [28] and then functionalized accordingly, yielding the GCI ethyl amide (for detailed synthesis routes, see Scheme S1 and Scheme S2 in Supporting Information File 1). The functionalization of the binding motifs GCP and GCI were performed since in the presence of the free guanidinium moiety the free carboxylic acid leads to a strong dimerization based on zwitterion formation at neutral pH, as described for GCP (*K*_dim_ >10^2^ in water) [29] as well as for a GCI derivative [30], which would interfere with the anion binding. Solid benzoic acid and RGD were purchased from Fluka Analytical and Sigma-Aldrich, respectively. Both chemicals were used without further purification. All liquid samples were prepared in 200 µM concentration in 6 mM BisTris buffer at pH 6.

## Supporting Information

DFT calculated normal modes with the corresponding wavenumbers and Raman activities of GCI ethyl amide and detailed synthesis routes for GCP and GCI ethyl amide.

File 1 DFT calculation results and detailed synthesis routes.
