# Supplementary material for: UV resonance Raman spectroscopy of the supramolecular ligand guanidiniocarbonyl indole (GCI) with 244 nm laser excitation
Source: Beilstein J Org Chem. 2020 Nov 27;16:2911–9. doi: 10.3762/bjoc.16.240 (PMC7705883; doi:10.3762/bjoc.16.240)
Supplement: File 1 — DFT calculation results and detailed synthesis routes. [file Beilstein_J_Org_Chem-16-2911-s001.pdf]

BEILSTEIN JOURNAL OF ORGANIC CHEMISTRY

Supporting Information

for

UV resonance Raman spectroscopy of the supramolecular ligand guanidiniocarbonyl indole (GCI) with 244 nm laser excitation

Tim Holtum, Vikas Kumar, Daniel Sebena, Jens Voskuhl and Sebastian Schlücker

Beilstein J. Org. Chem. **2020,** *16,* 2911–2919. doi:10.3762/bjoc.16.240

DFT calculation results and detailed synthesis routes

License and Terms: This is a supporting information file under the terms of the Creative Commons Attribution License (https://creativecommons.org/licenses/by/4.0). Please note that the reuse, redistribution and reproduction in particular requires that the author(s) and source are credited and that individual graphics may be subject to special legal provisions.
The license is subject to the *Beilstein Journal of Organic Chemistry* terms and conditions: (https://www.beilstein-journals.org/bjoc/terms)

Table S1 contains the theoretical vibrational spectrum of GCI ethyl amide in the single protonated form calculated at the B3LYP-D3/6-311++G(d,p) and B2PLYP-D3/G-311++G(d,p) level of theory employing the Gaussian 16 program package.[1] All normal modes together with their wavenumber values and Raman activities are listed.

Table S1: Calculated Raman spectrum of GCI ethyl amide in the single protonated form. Level of theory: B3LYP-D3/6-311++G(d,p) and B2PLYP-D3/G-311++G(d,p).

	B3LYP-D3		B2PLYP-D3	
Mode	Wavenumber [cm^{-1}]	Raman activity	Wavenumber [cm^{-1}]	Raman activity
1	19.48	1.650	22.56	1.857
2	36.23	2.199	37.39	2.455
3	41.13	2.368	42.92	2.573
4	46.44	3.180	46.89	2.895
5	60.56	1.389	60.66	1.324
6	73.76	1.081	70.48	0.948
7	86.71	1.478	87.15	1.367
8	119.78	0.187	111.99	0.926
9	140.21	2.560	131.24	2.393
10	169.97	1.194	168.00	0.993
11	203.40	0.577	196.19	0.736
12	218.96	4.264	217.09	6.302
13	254.10	6.136	229.33	8.079
14	257.77	0.205	250.85	2.605
15	272.11	2.126	253.84	0.567
16	276.35	0.812	272.22	0.647
17	297.11	2.149	290.47	4.896
18	312.57	6.133	293.77	6.009
19	328.64	1.071	324.94	0.981
20	362.50	1.251	354.45	0.818
21	387.30	2.255	380.69	2.587
22	407.20	4.323	401.72	7.257
23	421.46	2.672	406.81	2.777
24	459.21	2.524	434.76	1.154
25	471.14	1.205	456.26	1.678
26	481.28	11.412	465.27	1.040
27	484.08	9.582	483.51	21.404
28	502.38	5.236	493.68	5.021
29	533.87	2.244	522.52	1.435
30	584.91	2.130	572.50	3.959
31	589.17	4.033	585.82	4.151
32	603.06	7.906	596.31	7.477
33	614.22	4.644	611.77	4.061

34	666.93	29.087	655.34	5.020
35	689.16	6.086	663.70	21.355
36	705.35	8.833	671.54	12.714
37	723.32	1.879	693.78	8.293
38	739.28	0.573	723.94	0.789
39	746.57	2.597	725.57	3.438
40	774.83	16.557	769.92	13.046
41	784.71	4.784	780.33	3.424
42	817.87	11.777	801.62	5.651
43	819.32	15.825	814.89	24.967
44	846.48	7.412	829.19	8.057
45	855.83	2.907	838.54	0.944
46	866.71	1.123	844.94	3.677
47	887.68	6.525	885.87	6.720
48	898.35	45.927	896.60	69.450
49	931.17	15.989	926.07	2.193
50	962.74	0.969	927.82	21.909
51	970.20	22.033	967.93	24.435
52	1013.84	30.355	1019.52	23.145
53	1036.03	100.442	1034.13	72.298
54	1048.10	16.370	1047.45	11.570
55	1076.98	9.311	1076.81	9.791
56	1081.14	18.446	1082.25	12.124
57	1103.62	69.065	1106.82	113.040
58	1119.63	83.512	1113.93	27.110
59	1123.71	9.408	1127.13	6.597
60	1140.56	4.772	1138.65	30.739
61	1187.80	9.951	1183.44	9.895
62	1213.58	2.904	1205.34	2.310
63	1232.10	51.645	1229.34	30.302
64	1257.45	39.564	1256.48	40.743
65	1274.30	512.863	1274.30	282.310
66	1296.71	340.337	1295.87	429.881
67	1337.18	15.908	1342.55	69.137
68	1340.76	37.363	1344.11	18.332
69	1349.29	17.612	1352.06	95.731
70	1358.90	317.856	1358.26	236.080
71	1372.96	0.635	1376.22	0.552
72	1406.46	70.380	1405.92	142.007
73	1442.67	11.561	1448.84	11.242
74	1445.31	7.473	1452.04	7.271
75	1458.79	62.794	1458.73	29.875
76	1465.77	4.625	1462.90	127.130
77	1469.42	85.093	1473.34	11.261
78	1479.42	60.892	1474.55	75.608
79	1503.22	516.891	1501.26	629.622

80	1542.27	49.245	1541.42	42.261
81	1554.53	6.601	1555.04	10.480
82	1595.78	1 213.020	1592.44	1 018.563
83	1619.38	58.509	1619.18	67.806
84	1649.87	3.792	1655.08	6.583
85	1669.50	140.290	1656.61	120.609
86	1695.06	755.304	1688.11	923.291
87	2913.48	276.720	2928.48	194.390
88	2914.39	112.570	2933.32	158.137
89	2969.10	62.663	2989.04	66.470
90	2995.96	149.947	3013.22	61.384
91	2997.05	45.912	3015.20	108.538
92	3063.23	50.762	3071.45	51.893
93	3073.02	90.382	3081.17	86.851
94	3092.48	151.471	3099.40	155.391
95	3129.27	72.422	3133.62	72.461
96	3263.04	91.461	3285.21	124.052
97	3445.49	32.851	3446.00	40.914
98	3460.42	44.814	3456.11	55.482
99	3463.26	134.171	3467.97	122.943
100	3483.90	65.572	3485.52	66.280
101	3525.30	81.355	3534.45	68.328
102	3570.94	63.089	3580.53	55.943

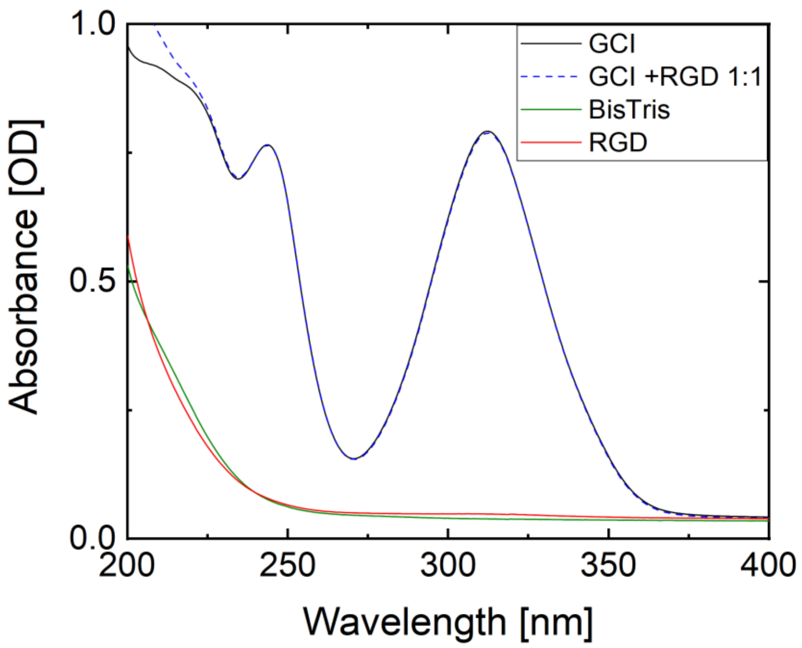

Figure S1: UV–vis absorption spectra of GCI, RGD, BisTrisBuffer and a 1:1 GCI–RGD mixture**.**

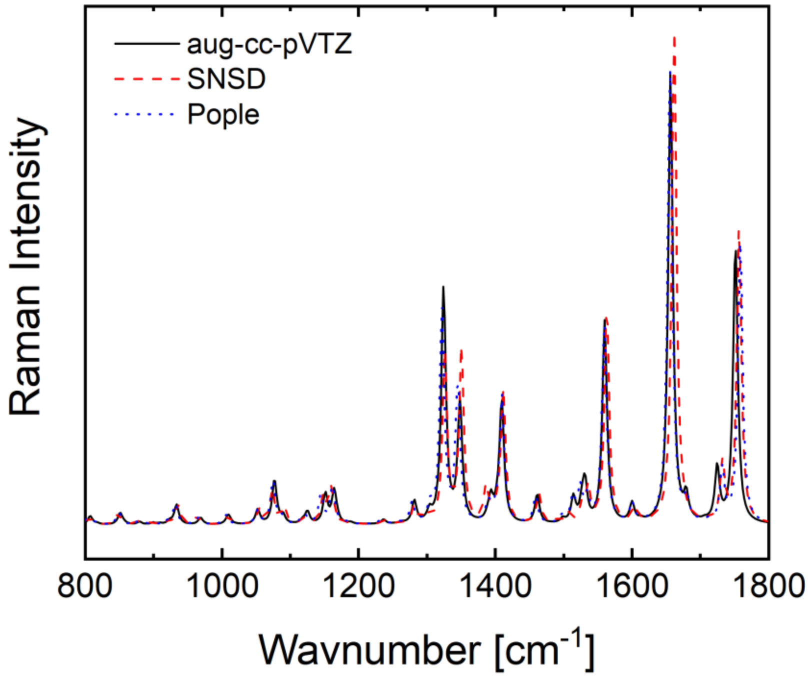

Figure S2: DFT calculated Raman spectrum of GCI ethyl amide plotted in the region 800–1800 cm^{-1} using the density functional B3LYP-D3 with three different basis sets: Pople & aug-cc-pVTZ & SNSD.

General information

All solvents were distilled before use. Millipore water was obtained with a TKA MicroPure ultrapure water system. All other commercially available reagents were used as obtained unless otherwise specified. The reactions were monitored by TLC on silica gel plates (Macherey-Nagel POLYGRAM SIL G/UV254) and spots were visualized by UV light (254 nm and 366 nm). Reversed phase column chromatography was performed with an Armen Instrument Spot Flash Liquid Chromatography MPLC apparatus with RediSep C-18 Reversed Phase columns. Lyophilisation was done with a Christ Alpha 1-4 LD plus freeze dryer. The melting points were obtained with a Büchi Melting-Point B-540 apparatus with open end glass capillary tubes. The melting points are not corrected. The IR spectra were measured on a Varian 3100 FT-IR Excalibur Series. The low resolution ESI mass spectra were recorded with a Bruker amaZon SL and the high resolution ESI mass spectra with a Bruker maXis 4G UHR-TOF. Analytical HPLC was performed on a Dionex HPLC apparatus that consisted of a P680 pump, an ASI-100 automated sample injector and an UVD 340U photodiode array detector with a YMC ODS-AQ column (column size: 150 × 3.0 mm, particle size: 5 µm, pore size: 12 nm). The NMR spectra were measured with Bruker DMX 300, AV NEO 400, DRX 500 or AVHD 600 spectrometers. All measurements were recorded at room temperature using DMSO-d_6 as solvent. The chemical shifts are relative to the signals of DMSO-d_6 (δ ^{1}H = 2.50 ppm and δ ^{13}C = 39.5 ppm). The apparent coupling constants are given in hertz (Hz). The description of the fine structure means: s = singlet, br. s = broad singlet, d = doublet, t = triplet, m = multiplet.

Synthesis

The GCI building block **I** was synthesized starting from commercially available methyl 3-amino-4-iodobenzoate (**A**) following a synthesis strategy inspired and adjusted from a previous work [2]. The building block **I** was further functionalized with ethyl amine to achieve GCI ethyl amide **2**.

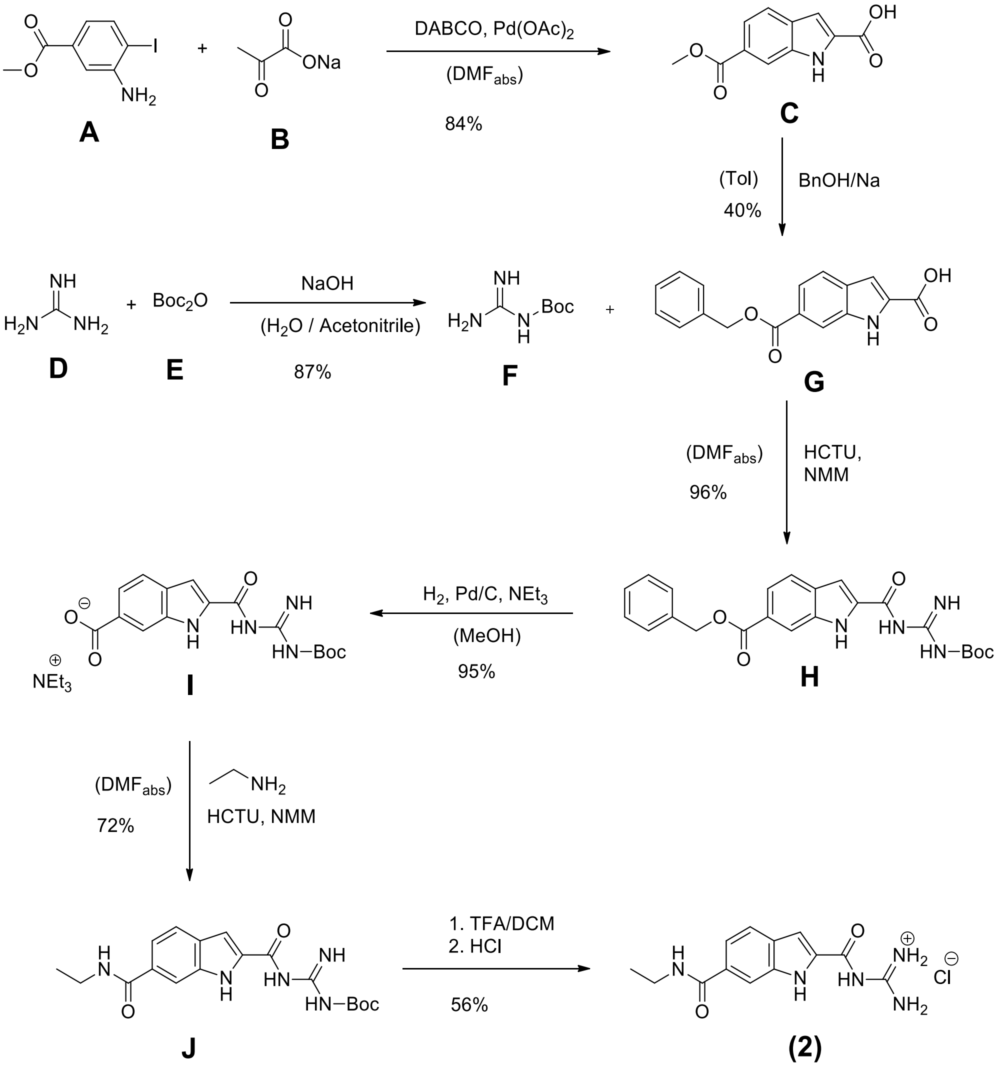

Scheme S1: Synthesis of the GCI ethyl amide **2**.

The GCP ethyl amide **1** [3] was synthesized starting from literature-known GCP building block **K** [4].

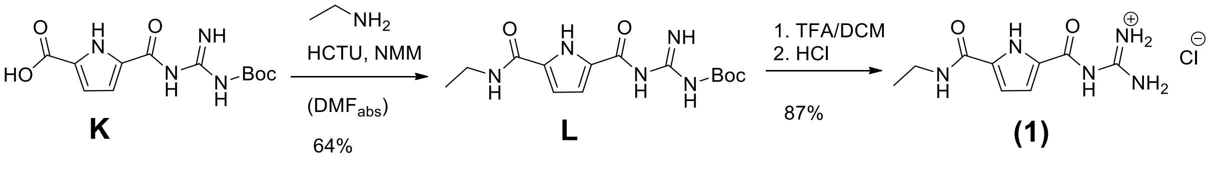

Scheme S2: Synthesis of the GCP ethyl amide **1**.

6-(Methoxycarbonyl)-1*H*-indole-2-carboxylic (C)

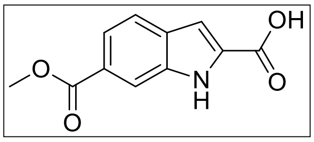

To freshly sublimed DABCO (6.074 g, 54.15 mmol, 3 equiv), sodium pyruvate (**B**, 5.959 g, 54,15 mmol, 3 equiv) and methyl 3-amino-4-iodobenzoate (**A**, 5 g, 18.05 mmol, 1 equiv), 260 mL degassed dry DMF was added under an argon atmosphere. Then, palladium(II) acetate (0.33 g, 1.47 mmol, 0.08 equiv) was added and the mixture was heated to 105 °C for 19 h. After cooling to room temperature, 150 mL water was added, the solution was acidified with 1 M HCl to pH 2 and extracted with ethyl acetate (5 × 150 mL). The combined organic layers were washed with brine (2 × 150 mL) and water (2 × 150 mL), dried ($MgSO_4$), and the solvent was evaporated in vacuo to obtain a brown solid. The crude product was purified by column chromatography (SiO_2, ethyl acetate/cyclohexane 2:1 + 1% acetic acid) to give compound **C** (3.321 g, 15.15 mmol, 84%) as yellow solid.

Molecular Formula: $C_{11}H_9NO_4$.

Molecular Mass: 219.193 g/mol.

^{1}H NMR (300 MHz, DMSO-d_6): δ [ppm] = 13.14 (s, 1H, COO*H*), 12.17 (s, 1H, N*H*), 8.11 (s, 1H, *H*-7), 7.75 (d, *J* = 8.5 Hz, 1H, *H*-5), 7.65 (dd, *J* = 8.5, 1.5 Hz, 1H, *H*-4), 7.16 (dd, *J* = 2.0, 0.8 Hz, 1H, *H*-3), 3.87 (s, 3H, C*H*$_3$).

^{13}C NMR (75 MHz, DMSO-d_6): δ [ppm] =166.79, 162.39, 131.61, 130.19, 125.07, 121.94, 120.94, 120.08, 114.51, 107.03, 51.93.

HR-MS: (pos. ESI, MeOH) m/z = 220.0616 ($[M+H]^+$, calc.: 220.0604).

FT-IR (ATR) ṽ [cm^{-1}] = 3648.66 (s), 2534.01 (w), 2159.88 (w), 2032.6 (w), 1683.55 (m), 1506.13 (m), 1438.64 (s), 1240 (m), 827.312 (s), 771.387 (s), 732.817 (s).

mp: 230 °C (decomposition).

N-tert-Boc-guanidine (F)

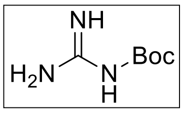

The reaction was performed as described in the literature [4]. A solution of *t*-Boc$_2$O (**E**, 12.0 g, 55.0 mmol, 1 equiv) in acetonitrile (100 mL) was added very slowly over 8 h at 0 °C under vigorous stirring to a mixture of guanidinium chloride (**D**, 26.3 g, 275 mmol, 5 equiv) in an aqueous sodium hydroxide solution (12.0 g, 0.3 mol NaOH in 50 mL water). The resulting suspension was stirred at room temperature for additional 20 h. The acetonitrile was evaporated in vacuo and then 100 mL water was added. The aqueous suspension was extracted with ethyl acetate (3 times with 100 mL). The combined organic phases were washed with brine (3 times with 100 mL), dried ($MgSO_4$), and evaporated in vacuo. The resulting white crystals were dried to yield 7.66 g (87%) of analytically pure guanidine **F**.

Molecular Formula: $C_6H_{13}N_3O_2$.

Molecular Mass: 159.186 g/mol.

^{1}H NMR (300 MHz, DMSO-d_6): δ [ppm] = 6.88 (br.s 4H, N*H*), 1.33 (s, 9H, Boc-*H*).

^{13}C NMR (75 MHz, DMSO-d_6): δ [ppm] = 28.19, 75.49, 162.62, 163.25.

HR-MS: (pos. ESI, MeOH) m/z = 160.1081 ([M+H]$^+$, calc.: 160.1081).

FT-IR (ATR) ṽ [cm^{-1}] = 3408 (s), 1650 (s), 1540 (s), 1450 (m), 1311 (s), 1253 (m), 1142 (s), 1066 (s), 950 (w), 806 (m).

mp: 165 °C (decomposition).

6-((Benzyloxy)carbonyl)-1*H*-indole-2-carboxylic acid (G)

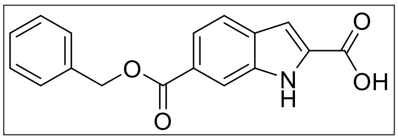

A sodium benzylate solution, prepared from sodium (0.603 g, 26.33 mmol, 5 equiv) in dry benzyl alcohol (300 mL), was added to a solution of carboxylic acid **C** (1.071 g 4.88 mmol, 1 equiv) in dry benzylalcohol (48 mL) and dry toluene (42 mL) under argon. The resulting dark brown solution was stirred at 95 °C for 4 h. After cooling to room temperature, 1 M hydrochloric acid (26 mL) and water (60 mL) was added. The solution was extracted with chloroform (3 × 100 mL) and the solvent evaporated in vacuo. The crude product was purified by flash chromatography (RP-18 MeOH/H_2O, 40% MeOH to 100% MeOH, gradient) to give product **G** (0.589 g, 2.03 mmol, 40%) as yellow solid.

Molecular Formula: $C_{17}H_{13}NO_4$.

Molecular Mass: 295.289 g/mol.

^{1}H NMR (300 MHz, DMSO-d_6): δ [ppm] = 13.23 (s, 1H, COO*H*), 12.13 (s, 1H, N*H*), 8.15 (s, 1H, *H*-7), 7.76 (d, *J* = 8.5 Hz, 1H, *H*-5), 7.68 (dd, *J* = 8.5, 1.4 Hz,1H, *H*-4), 7.52 – 7.33 (m, 5H, Cbz-Ar-*H*), 7.19 – 7.11 (m, 1H, *H*-3), 5.37 (s, 2H, Cbz-CH_2).

^{13}C NMR (75 MHz, DMSO-d_6): δ [ppm] = 166.15, 162.33, 136.27, 131.71, 130.31, 128.49, 128.05, 127.95, 124.99, 122.02, 120.14, 114.64, 107.03, 65.99.

HR-MS: (pos. ESI, MeOH) m/z = 296.0920 ([M+H]$^+$, calc.: 296.0917).

FT-IR (ATR) ṽ [cm^{-1}] = 3853.08 (s), 3748.94 (s), 3675.66 (s), 3648.66 (s), 3253.32 (w), 2528.22 (w), 2159.88 (w), 2034.53 (w), 1673.91 (m), 1324.86 (m), 1241.93 (m), 1207.22 (m).

mp: 209 °C (decomposition).

Benzyl 2-((*N*-(*tert*-butoxycarbonyl)carbamimidoyl)carbamoyl)-1H-indole-6-carboxylate (H)

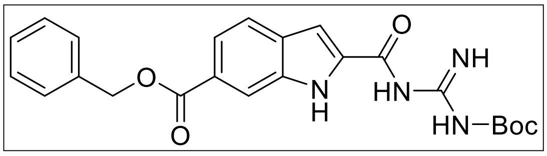

A mixture of the benzyl ester **G** (0.358 g, 1.256 mmol, 1 equiv), HCTU (1.0392 g, 2.512 mmol, 2 equiv), and NMM (0.5082 g, 5.024 mmol, 4 equiv) was stirred in DMF abs. (15 mL) at room temperature for 15 min. Then, *t*-Boc-guanidine **F** (0.299 g, 1.884 mmol, 1.5 equiv) was added and the resulting solution stirred at 40 °C. After 19 h the solution was poured into vigorously stirred water (150 mL) at 0 °C. A slightly yellow solid precipitated. The product was filtered, washed with cold water, and dried in vacuo, yielding the product **H** as slightly yellow solid (0.528 g, 1.21 mmol, 96%).

Molecular Formula: $C_{23}H_{24}N_4O_5$.

Molecular Mass: 436.47 g/mol.

^{1}H NMR (400 MHz, DMSO-d_6): δ [ppm] = 11.82 (s, 1H, N*H*), 10.99 (s, 1H, N*H*), 9.48 (s, 1H, N*H*), 8.63 (s, 1H, N*H*), 8.17 (s, 1H, *H*-7), 7.73 (d, J = 8.5 Hz, 1H, *H*-5), 7.65 (dd, *J* = 8.5, 1.4 Hz, 1H, *H*-4), 7.51 – 7.33 (m, 5H, Cbz-Ar-*H*), 7.18 (s, 1H, *H*-3), 5.36 (s, 2H, Cbz-CH_2), 1.48 (s, 9H, Boc-CH_3).

^{13}C NMR (101 MHz, DMSO-d_6): δ [ppm] = 166.33, 158.64, 136.42, 136.16, 130.72, 128.55, 128.09, 128.00, 121.88, 119.96, 114.62, 105.64, 65.96, 27.73.

HR-MS: (pos. ESI, MeOH) m/z = 437.1815 ($[M+H]^+$, calc.: 437.1819).

FT-IR: (ATR) ṽ [cm-1] = 3853.08 (s), 3748.94 (s), 3675.66 (s), 3648.66 (s), 2524.36 (w), 2159.88 (w), 2030.68 (w), 1700.91 (m), 1617.98 (m), 1558.2 (m), 1540.85 (m), 1496.49 (m), 1191.79 (m), 1145.51 (m), 1085.73 (m).

mp: 270 °C.

GCI building block (I)

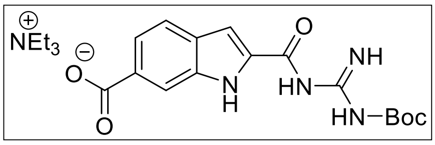

A mixture of the benzyl ester **H** (0.528 g, 1.21 mmol, 1 equiv), 10% Pd/C (30 mg) in 150 mL methanol, and 5 mL triethylamine was vigorously stirred under a hydrogen atmosphere for 16 h. The resulting solution was filtered through a folded filter and washed with methanol/triethylamine. The solvent was removed under reduced pressure yielding the GCI building block **I** as off-white solid (0.516 g, 1.15 mmol, 95%).

Molecular Formula: $C_{22}H_{33}N_5O_5$.

Molecular Mass: 447.528 g/mol.

^{1}H NMR (400 MHz, DMSO-*d*$_6$): δ [ppm] = 11.76 (s, 1H, N*H*), 9.46 (s, 1H, N*H*), 8.63 (s, 1H, N*H*), 8.09 (d, J = 0.8 Hz, 1H, *H*-7), 7.68 – 7.59 (m, 2H, *H*-5, *H*-4), 7.17 (s, 1H, *H*-3), 2.65 (q, J = 7.2 Hz, 6H, NEt$_3$-C*H*$_2$), 1.48 (s, 9H, Boc-C*H*$_3$), 1.02 (t, J = 7.2 Hz, 9H, NEt$_3$-C*H*$_3$).

^{13}C NMR (101 MHz, DMSO-*d*$_6$): δ [ppm] = 168.79, 158.59, 136.43, 129.77, 121.21, 120.51, 114.37, 105.70, 81.50, 45.37, 27.75, 10.48.

HR-MS: (pos. ESI, MeOH) m/z = 347.1351 ([M+H]$^+$, calc.: 347.1350).

FT-IR (ATR) ṽ [cm^{-1}] = 3648.66 (s), 2979.48 (w), 2159.88 (w), 1718.26 (s), 1540.85 (m), 1496.49 (m), 1365.35 (m), 1321 (w), 1238.08 (w), 1147.44 (w), 790.671 (s), 746.317 (s).

mp: 138 °C.

Boc-GCI ethyl amide J

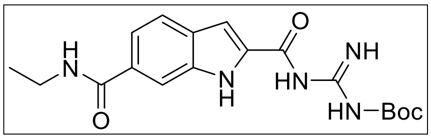

The GCI building block **I** (79 mg, 0.223 mmol, 1 equiv) and HCTU (184.2 mg, 0.446 mmol, 2 equiv) was dissolved in DMF abs. (10 mL) and NMM (90.23 mg, 0.892 mmol, 4 equiv) was added. After stirring the solution for 20 min at room temperature ethylamine (15 mg, 0.335 mmol, 1.5 equiv) was added and the resulting solution was stirred at room temperature for 17 h. The solvent was removed under reduced pressure and the crude product was purified by flash chromatography (RP-18 MeOH/H_2O, 20% MeOH to 100% MeOH, gradient) to give amide **J** (60.1 mg, 0.161 mmol, 72%) as white solid.

Molecular Formula: $C_{18}H_{23}N_5O_4$.

Molecular Mass: 373.406 g/mol.

^{1}H NMR (300 MHz, DMSO-*d*$_6$): δ [ppm] = 11.73 (s, 1H, N*H*), 10.96 (s, 1H, N*H*), 9.43 (s, 1H, N*H*), 8.61 (s, 1H, N*H*), 8.41 (t, *J* = 5.5 Hz, 1H, *H*-7), 7.95 (s, 1H, *H*-3), 7.65 (d, *J* = 8.4 Hz, 1H, *H*-5), 7.52 (dd, J = 8.5 Hz, 1H, *H*-4), 7.17 (s, 1H, N*H*), 3.35 – 3.24 (m, 2H, C*H*$_2$), 1.47 (s, 9H, Boc-C*H*$_3$), 1.13 (t, *J* = 7.2 Hz, 3H, C*H*$_3$).

^{13}C NMR (300 MHz, DMSO-*d*$_6$): δ [ppm]= 159.58, 158.79, 155.42, 132.97, 125.22, 115.90, 112.17, 33.62, 14.58.

HR-MS: (pos. ESI, MeOH) m/z = 374.1826 ([M+H]$^+$, calc.: 374.1823).

FT-IR (ATR) ṽ [cm-1]: 3357.46 (w), 2979.48 (w), 1725.98 (w), 1625.7 (m), 1546.63 (m), 1511.92 (m), 1369.21 (w), 1326.79 (m), 1241.93 (s), 1147.44 (s), 844.669 (m), 752.102 (w), 632.537 (m), 609.396 (m).

mp: 124 °C (decomposition).

GCI ethyl amide 2

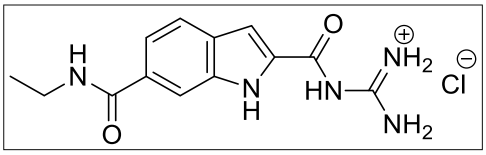

To a solution of Boc-GCI ethyl amide **J** (221 mg, 0.592 mmol, 1 equiv) in DCM/TFA (15 mL each), was added and the solution was stirred at room temperature for 3 h. The solvent was evaporated in vacuo to receive the off-white crude product, which was purified by flash chromatography (RP 18 MeOH/H_2O + 0.1% TFA, 10% MeOH + 0.1% TFA to 100% MeOH + 0.1% TFA, gradient) and treated several times with 1 M HCl with respective solvent removal to give the chloride salt **2** (103 mg, 0.333 mmol, 56%) as white solid with a purity of 97% (HPLC).

Molecular Formular: $C_{13}H_{16}ClN_5O_2$.

Molecular Mass: 309.751 g/mol.

^{1}H NMR (600 MHz, DMSO-d_6): δ [ppm] = 12.36 (m, 2H, indole-N*H*, amide-N*H*), 8.76 (s, 2H, guanidine-N*H*), 8.54 (m, 3H, guanidine-N*H*), 7.99 (s, 1H, *H*-7), 7.95 (d,1H, *J* = 1.4 Hz, *H*-3), 7.76 (d, 1H, *J* = 8.5 Hz, *H*-5), 7.59 (dd, *J* = 8.5, 1.3 Hz, 1H, *H*-4), 3.33 – 3.27 (m, 2H, CH_2), 1.13 (t, 3H, *J* = 7.2 Hz, CH_3).

^{13}C NMR (151 MHz, DMSO-d_6): δ [ppm] = 166.32, 161.06, 155.46, 137.37, 132.07, 130.24, 128.25, 122.17, 119.40, 112.44, 108.00, 34.14, 14.87.

HR-MS: (pos. ESI, MeOH) m/z = 274.1318 ($[M+H]^+$, calc.: 274.1299)

FT-IR (ATR) ṽ [cm^{-1}] = 3097.12 (w), 2159.88 (w), 2032.6 (w), 1683.55 (m), 1635.34 (m), 1606.41 (m), 1560.13 (m), 1498.42 (s), 1448.28 (s), 1413.57 (s), 1375 (s), 1322.93 (m), 1216.86 (w), 1081.87 (s), 836.955 (s), 727.032 (m), 632.537 (w).

mp: 189 °C.

Boc-GCP-ethyl amide L

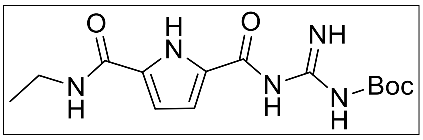

GCP building block **K** (5 g, 12.6 mmol, 1 equiv), HCTU (10.42 g, 25.2 mmol, 2 equiv) and NMM (5.10 g, 50.4 mmol, 4 equiv) were dissolved in dry DMF (200 mL). After 15 min ethylamine [2 M in THF] (9.5 mL, 18.9 mmol, 1.5 equiv) was added and the reaction mixture was stirred at room temperature for 16 h, extracted with chloroform (5 × 100 mL) and dried in vacuo. The crude product was purified by flash chromatography (RP-18 MeOH/H_2O, 10% MeOH to 100% MeOH, gradient) to give the product **L** (2.62 g, 8.11 mmol, 64%) as off-white solid.

Molecular Formula: $C_{14}H_{21}N_5O_4$.

Molecular Mass: 323.348 g/mol.

^{1}H NMR (300 MHz, DMSO-*d*$_6$): δ [ppm] = 11.24 (s, 1H, N*H*), 10.85 (s, 1H, N*H*), 9.32 (s, 1H, N*H*), 8.57 (s, 1H, N*H*), 8.33 (s, 1H, N*H*), 6.81 (s, 1H, pyrrole-C*H*), 6.75 (d, J = 3.8 Hz, 1H, pyrrole-C*H*), 3.25 (q, *J* = 7.1, 5.6 Hz, 2H, C*H*$_2$), 1.45 (s, 9H, Boc-C*H*$_3$), 1.11 (t, *J* = 7.2 Hz, 3H, C*H*$_3$).

^{13}C NMR (151 MHz, DMSO-*d*$_6$): δ [ppm] = 159.37, 158.43, 111.50, 48.60, 33.54, 27.77, 14.78.

HR-MS: (pos. ESI, MeOH) m/z = 324.1666 ([M+H]$^+$, calc.: 324.1666).

FT-IR (ATR) ṽ [cm^{-1}]: 3748.94 (s), 3648.66 (s), 3318.89 94. (w), 2977.55 (w), 2159.88 (w), 2030.68 (w), 1718.26 (s), 1621.84 (m), 1540.85 (m), 1455.99 (s), 1367.28 (s), 1286.29 (m), 1238.08 (w), 1141.65 (w), 842.74 (s), 748.245 (m);

mp: 138 °C.

GCP ethyl amide 1

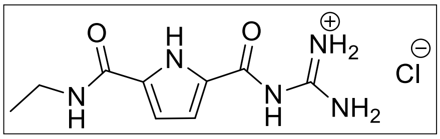

Boc-GCP-ethyl amide **L** (101 mg, 0.308 mmol, 1 equiv) was dissolved in dichlormethane (2 mL). TFA (2 mL) was added and the reaction mixture was stirred at room temperature for 3 h. The solvent was removed under reduced pressure and the crude product was treated with 1 M HCl. The solvent was removed to receive GCP ethyl amide **1** as white solid (90 mg, 0.267 mmol, 87%) with a purity of 95% (HPLC).

Molecular Formula: $C_9H_{14}ClN_5O_2$.

Molecular Mass: 259.693 g/mol.

^{1}H NMR (600 MHz, DMSO-*d*$_6$): δ [ppm] = 12.32 (s, 1H, pyrrole-N*H*), 11.32 (s, 1H, amide-N*H*), 8.55 – 8.12 (m, J = 11.2, 5.1 Hz, 5H, guanidine-N*H*), 7.16 (dd, J = 3.9, 2.3 Hz, 1H, pyrrole-C*H*), 6.85 (dd, J = 3.8, 2.4 Hz, 1H, pyrrole-C*H*), 3.33 – 3.21 (m, 2H, C*H*$_2$), 1.12 (t, J = 7.2 Hz, 3H, C*H*$_3$).

^{13}C NMR (151 MHz, DMSO-*d*$_6$): δ [ppm] = 158.81, 155.03, 132.89, 125.22, 115.50, 112.09, 33.63, 14.58.

HR-MS: (pos. ESI, MeOH) m/z = 224.1148 ([M+H]$^+$, calc.: 224.1142).

FT-IR: (ATR) ṽ [cm^{-1}] = 3309.21 (w), 2159.88 (s), 1685.48 (w), 1633.41 (m), 1558.2 (m), 1473.35 (s), 1292.07 (s), 1203.36 (s), 1064.51 (s), 804.171 (s), 746.317 (m), 698.105 (m), 609.396 (m).

mp: 234 °C (decomposition).

NMR Spectra

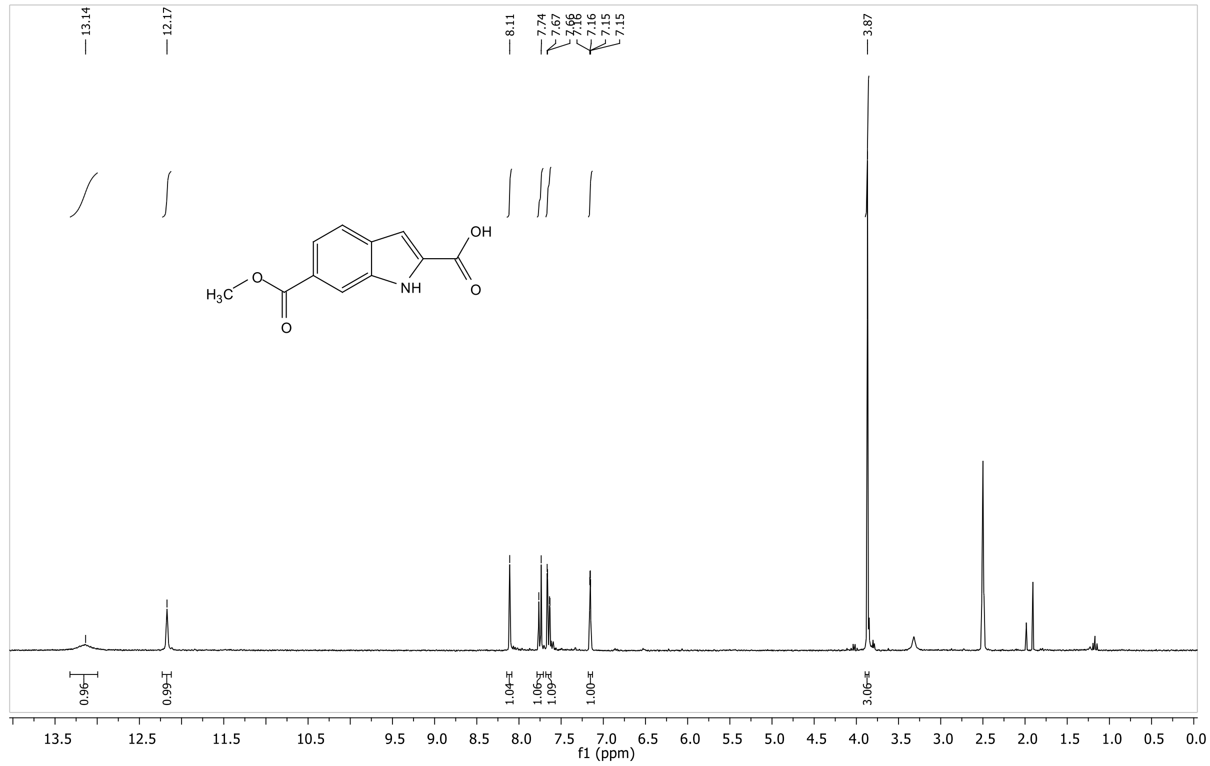

Figure S3: ^{1}H NMR spectrum of **C** (300 MHz, DMSO-*d*6).

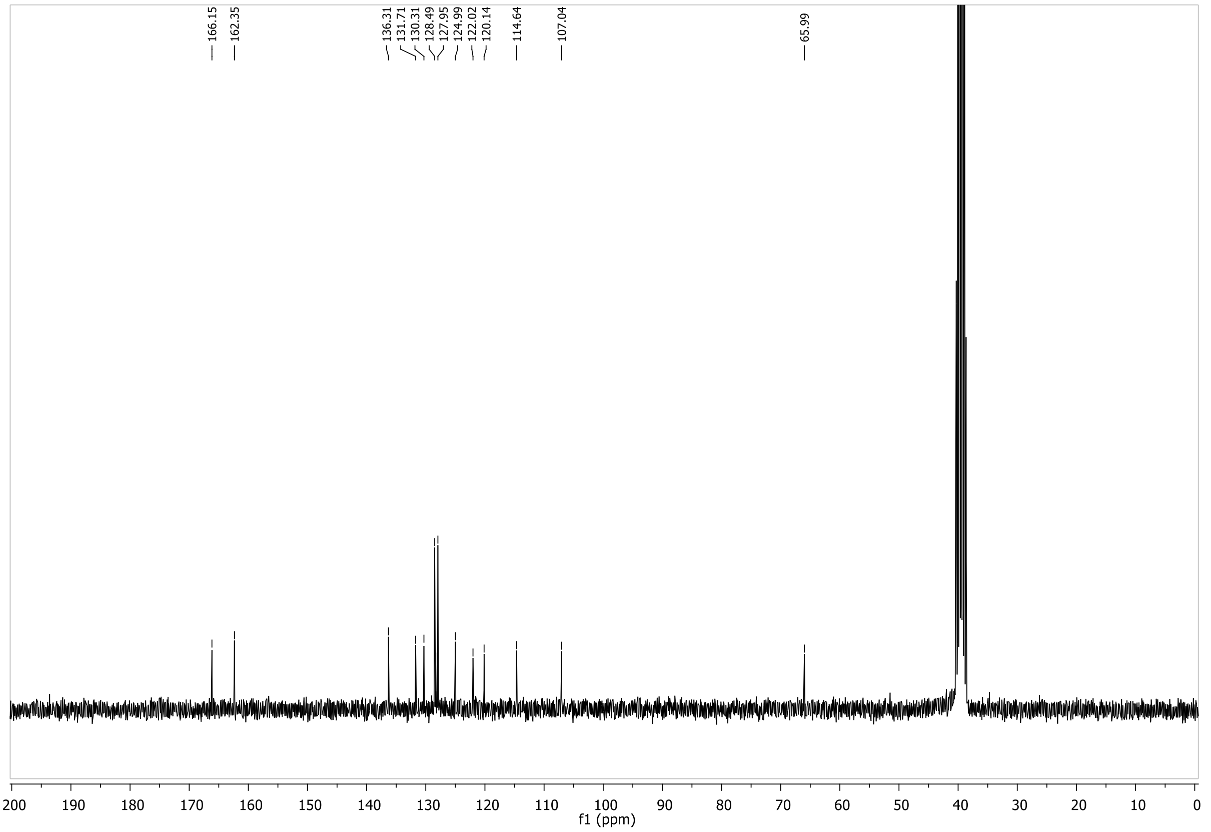

Figure S4: ^{13}C NMR spectrum of **C** (75 MHz, DMSO-*d*6).

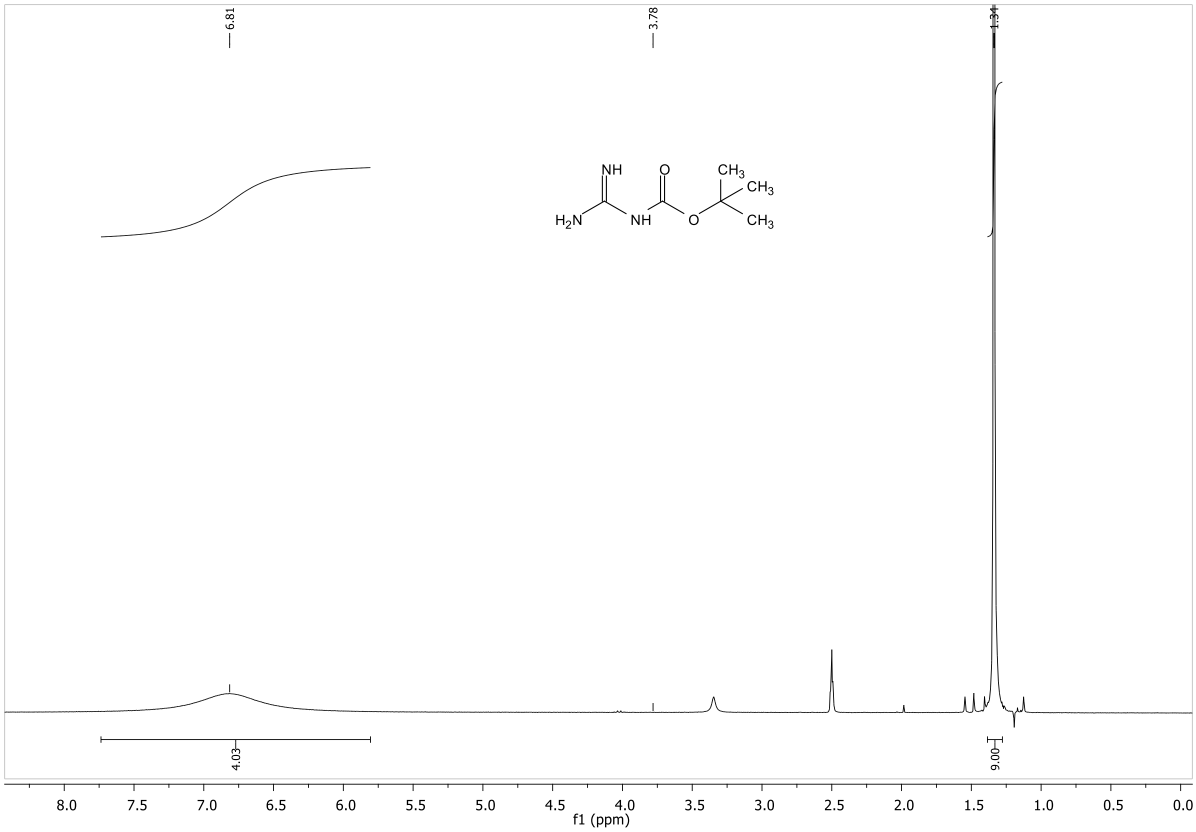

Figure S5: ^{1}H NMR spectrum of **F** (300 MHz, DMSO-*d*6).

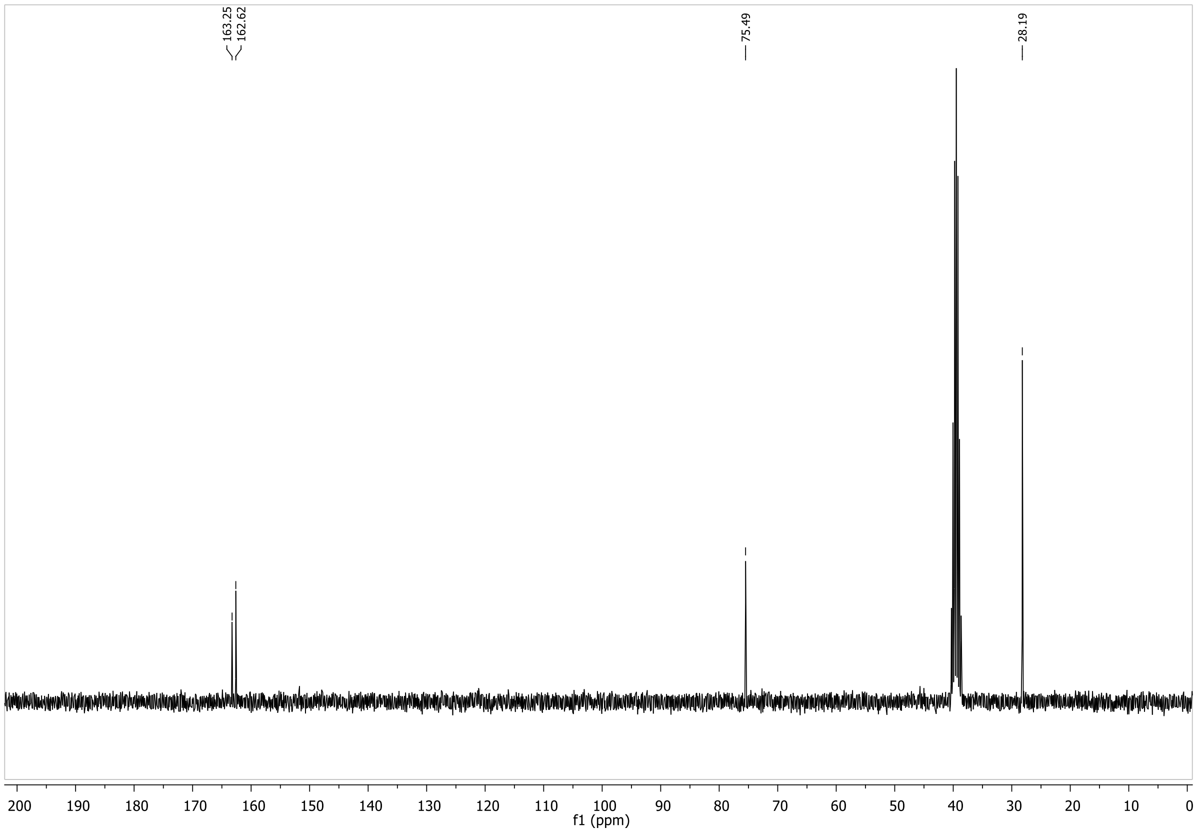

Figure S6: ^{13}C NMR spectrum of **F** (75 MHz, DMSO-*d*6).

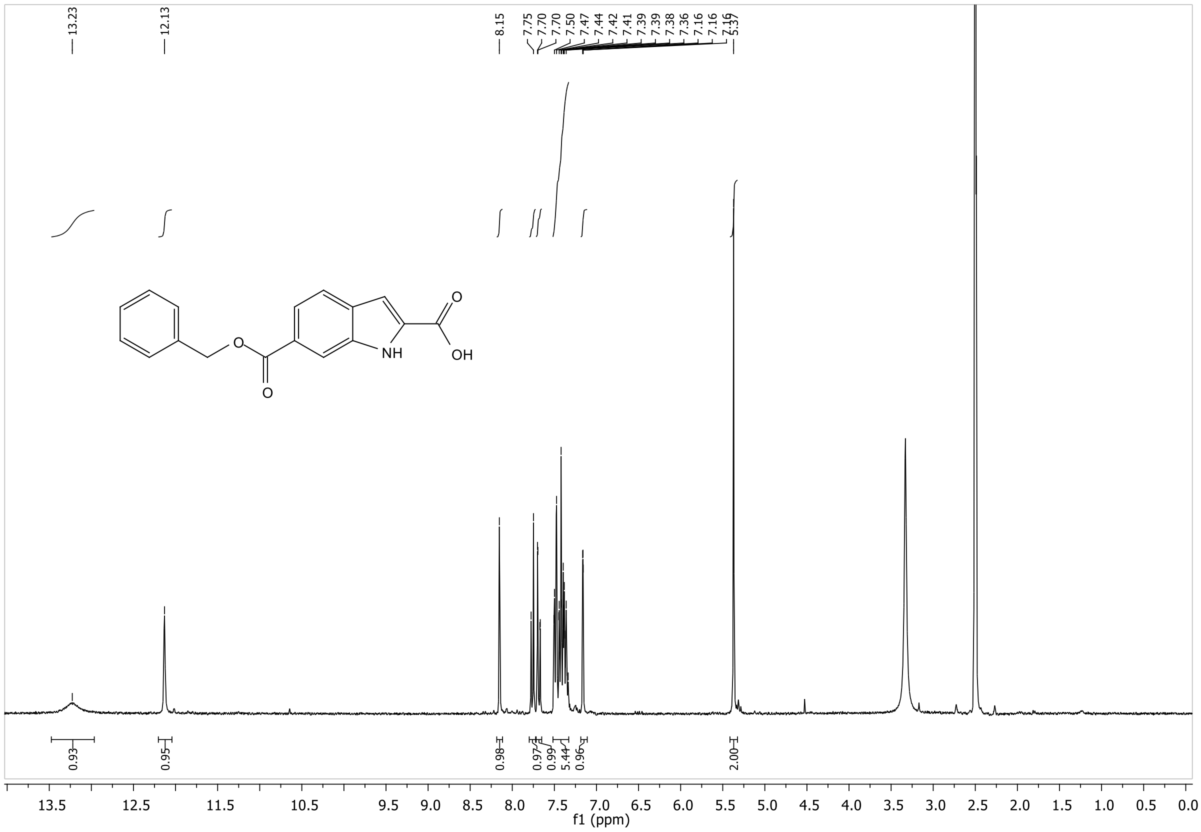

Figure S7: ^{1}H NMR spectrum of **G** (300 MHz, DMSO-d_6).

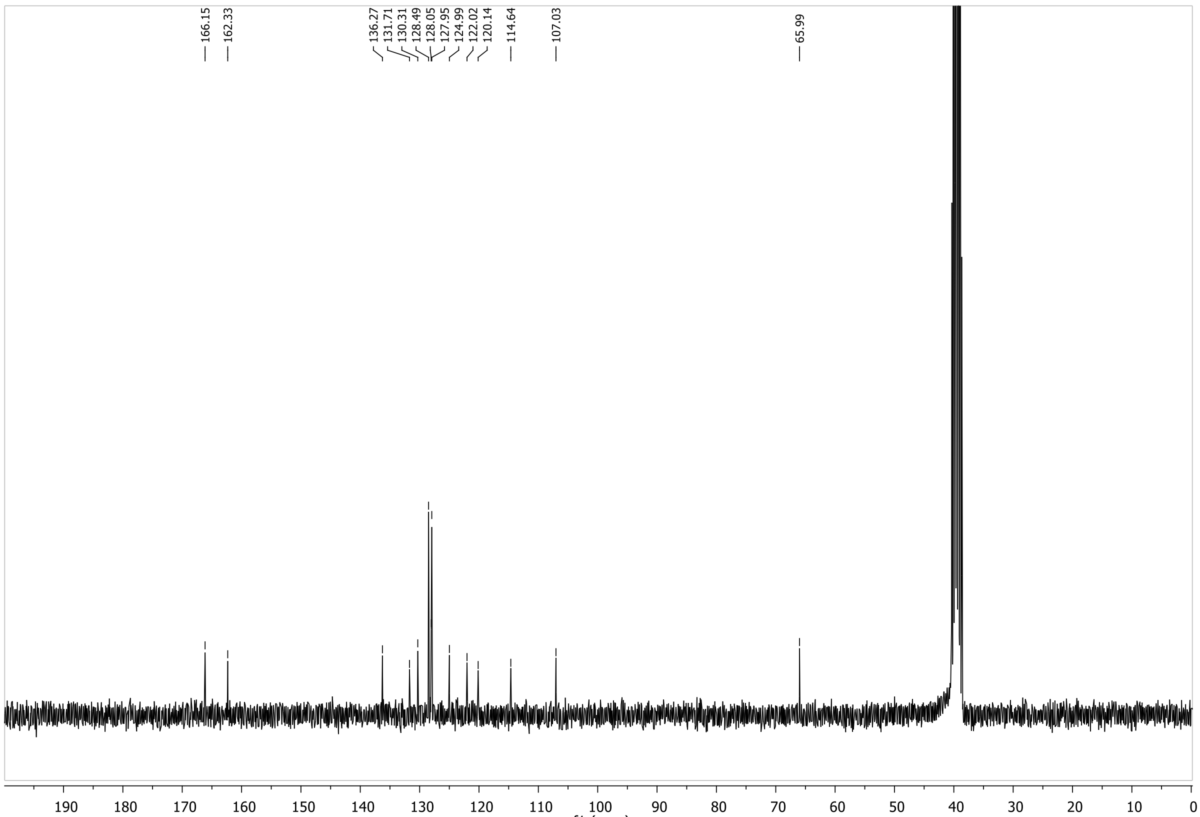

Figure S8: ^{13}C NMR spectrum of **G** (75 MHz, DMSO-d_6).

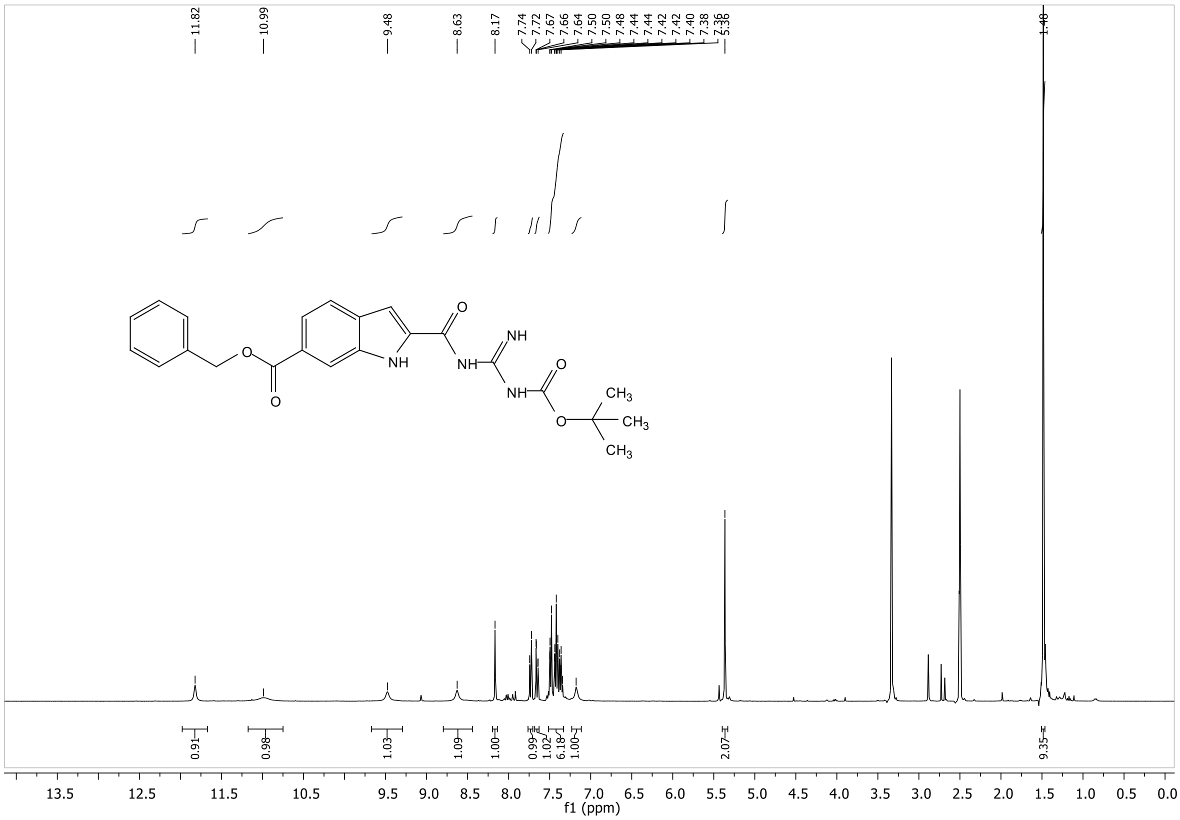

Figure S9: ^{1}H NMR spectrum of **H** (400 MHz, DMSO-d_6).

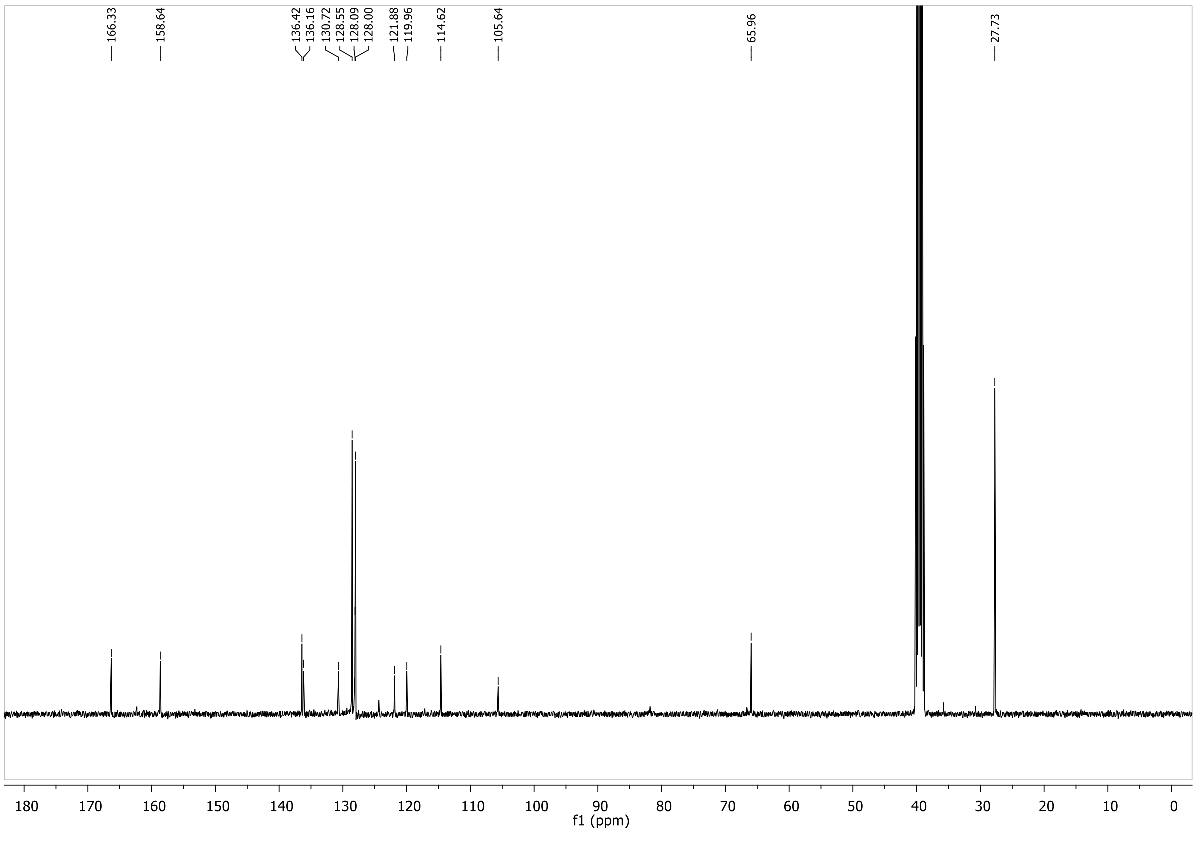

Figure S10: ^{13}C NMR spectrum of **H** (101 MHz, DMSO-d_6).

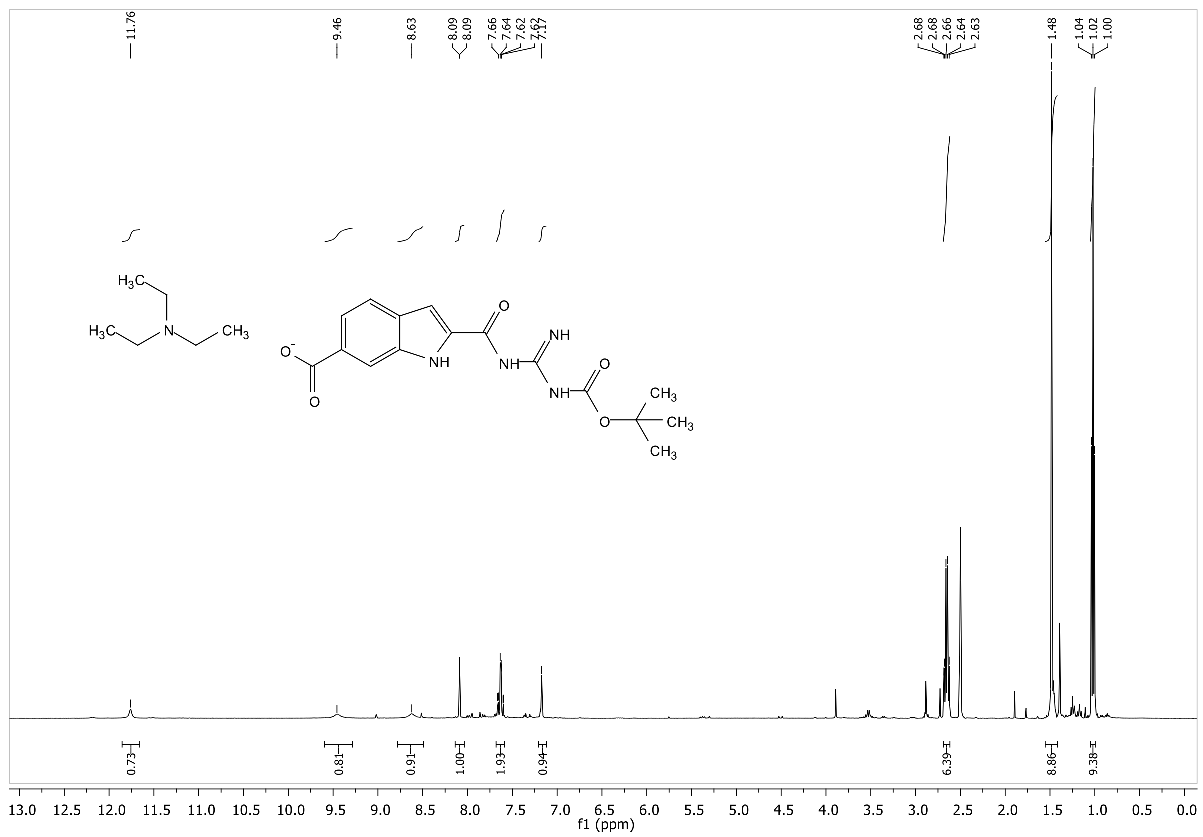

Figure S11: ^{1}H NMR spectrum of **I** (400 MHz, DMSO-*d*$_6$).

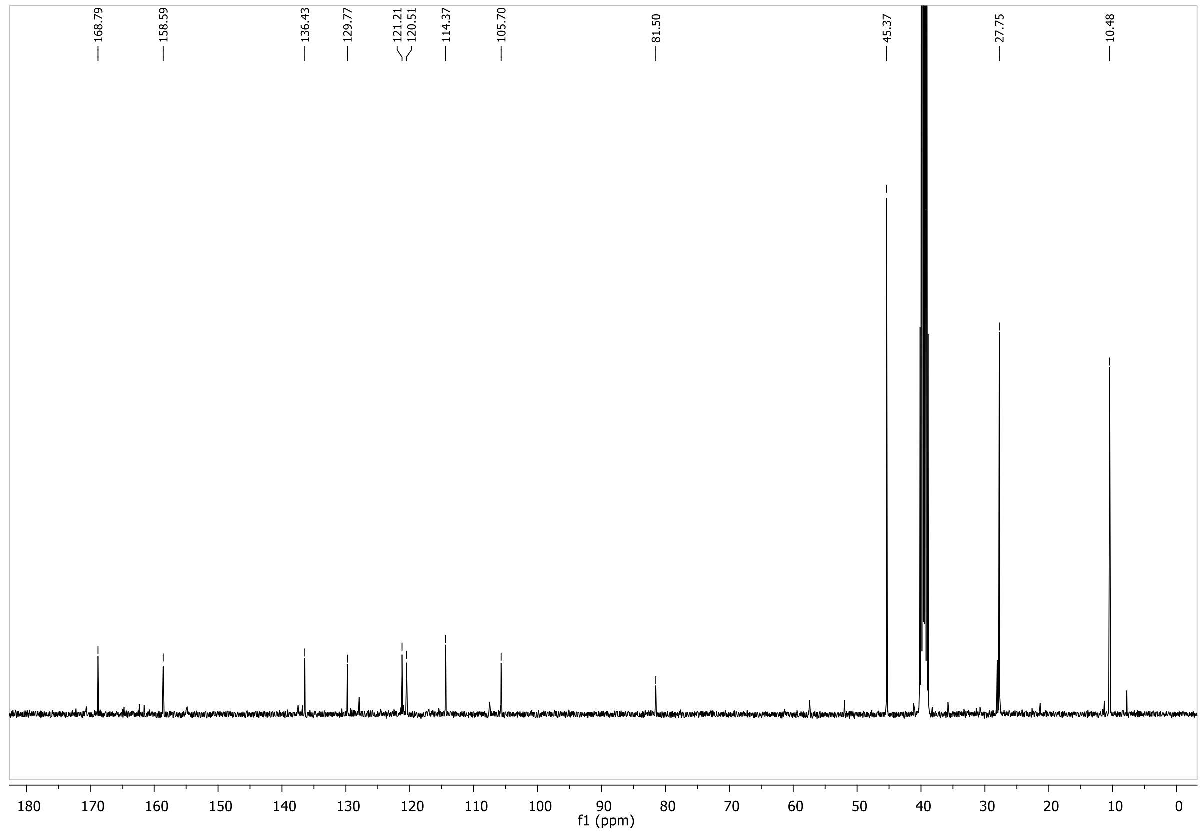

Figure S12: ^{13}C NMR spectrum of **I** (101 MHz, DMSO-*d*$_6$).

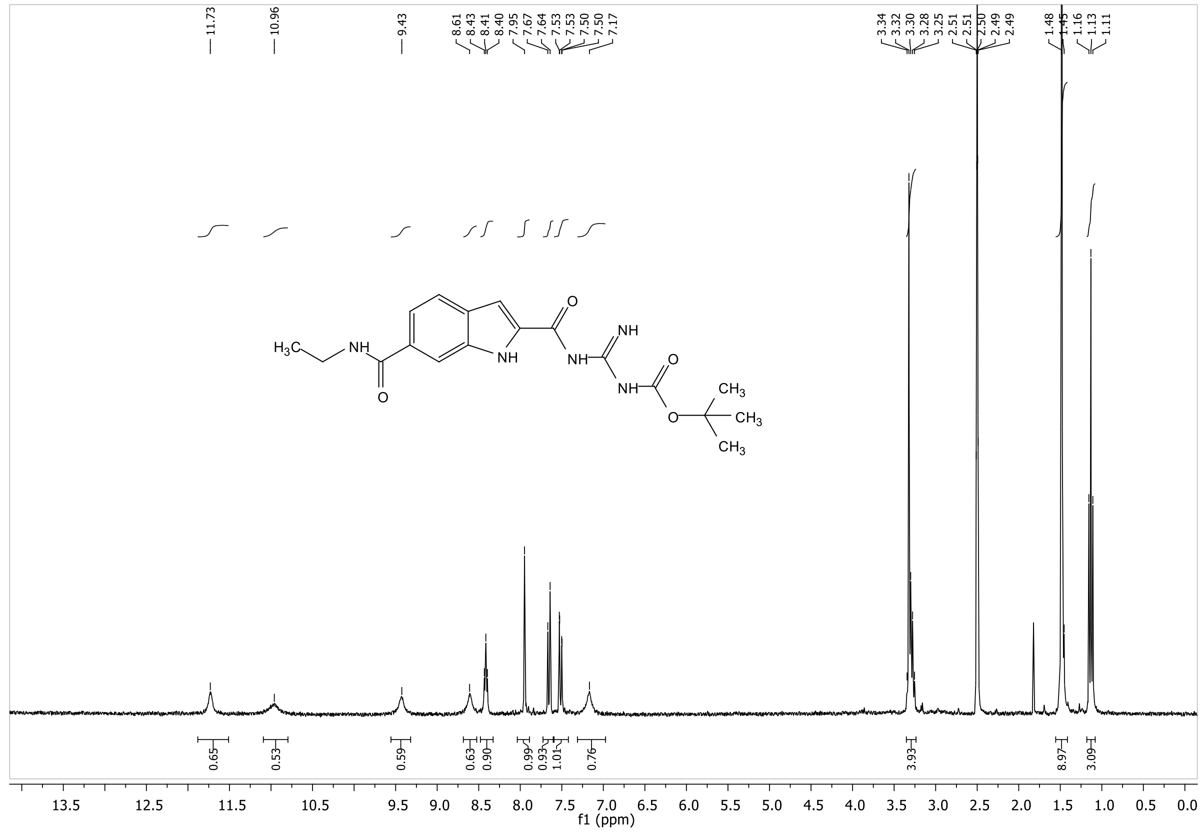

Figure S13: ^{1}H NMR spectrum of **J** (300 MHz, DMSO-d_6).

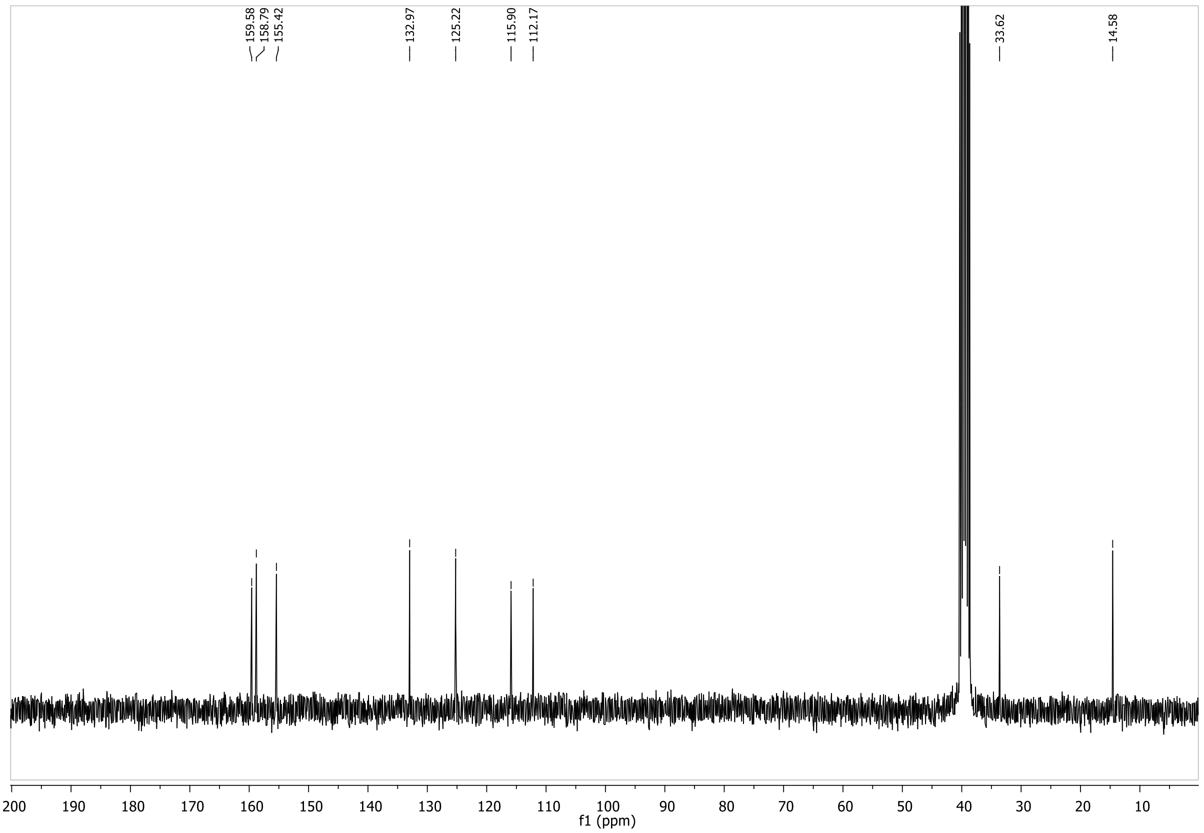

Figure S14: ^{13}C NMR spectrum of **J** (75 MHz, DMSO-d_6).

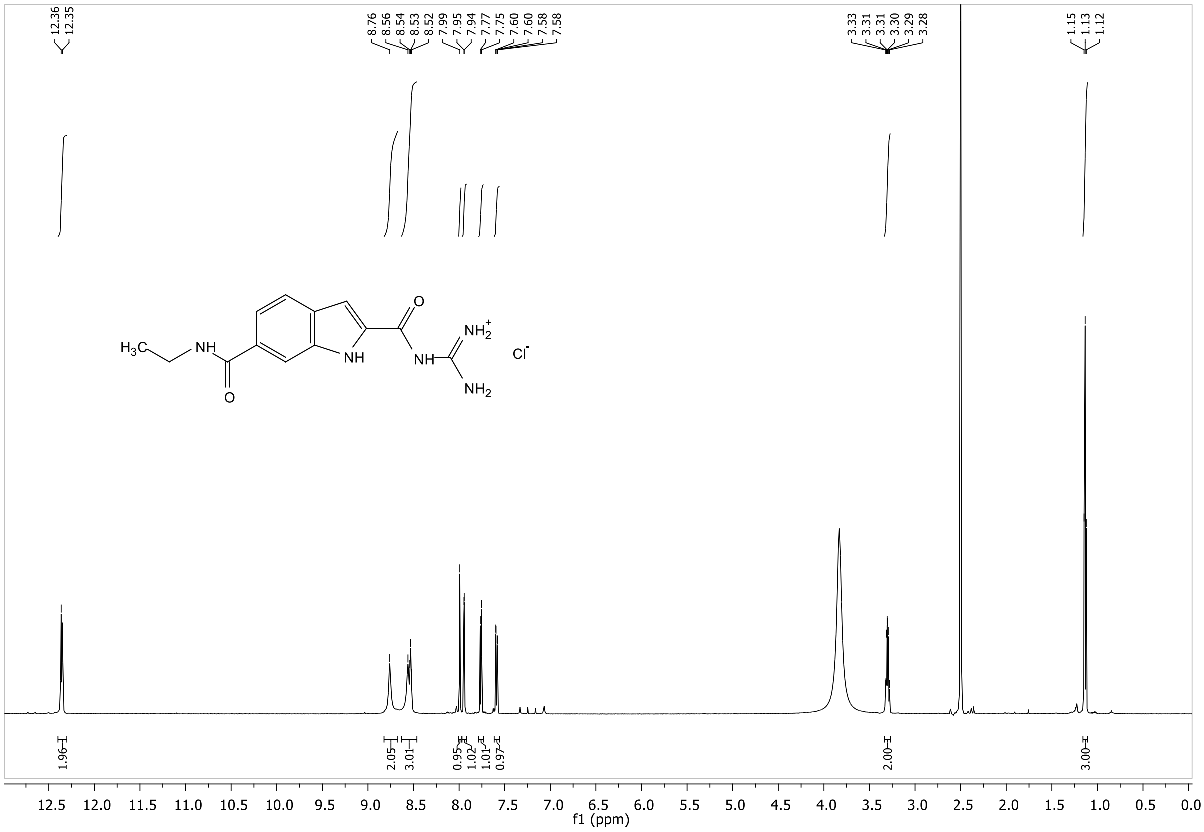

Figure S15: ^{1}H NMR spectrum of **2** (600 MHz, DMSO-*d*6).

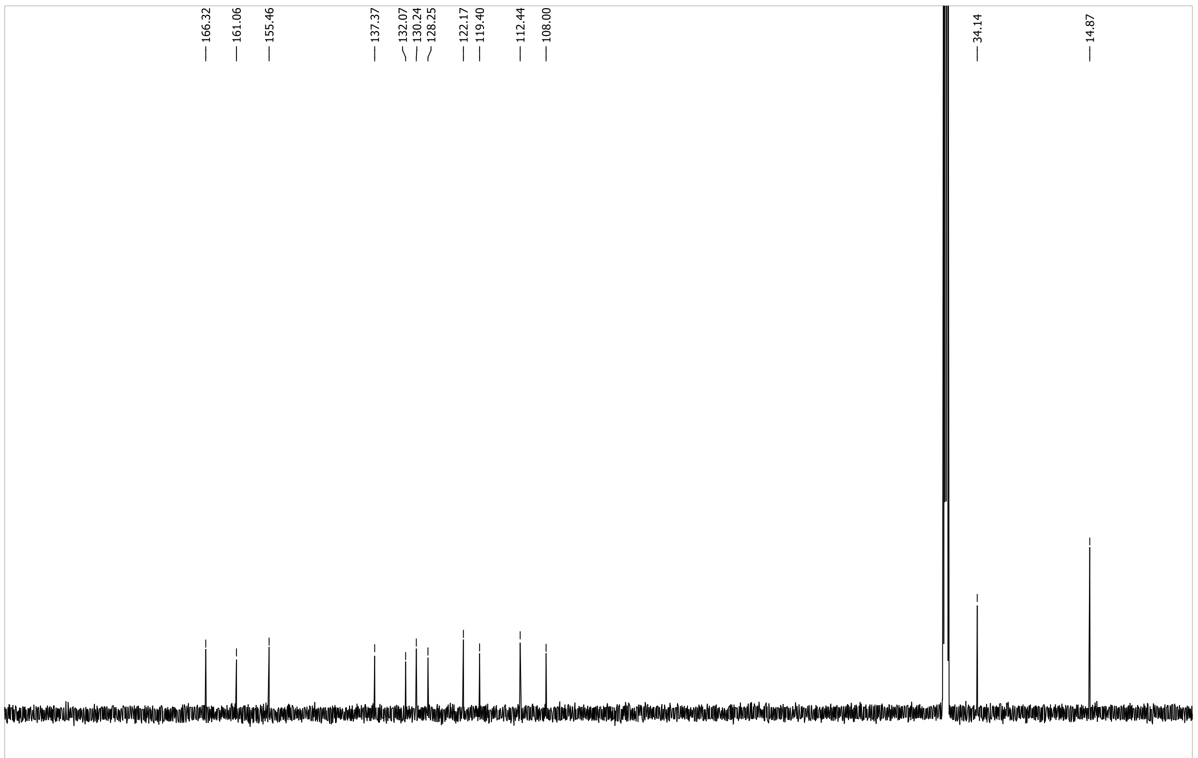

Figure S16: ^{13}C NMR spectrum of **2** (151 MHz, DMSO-*d*6).

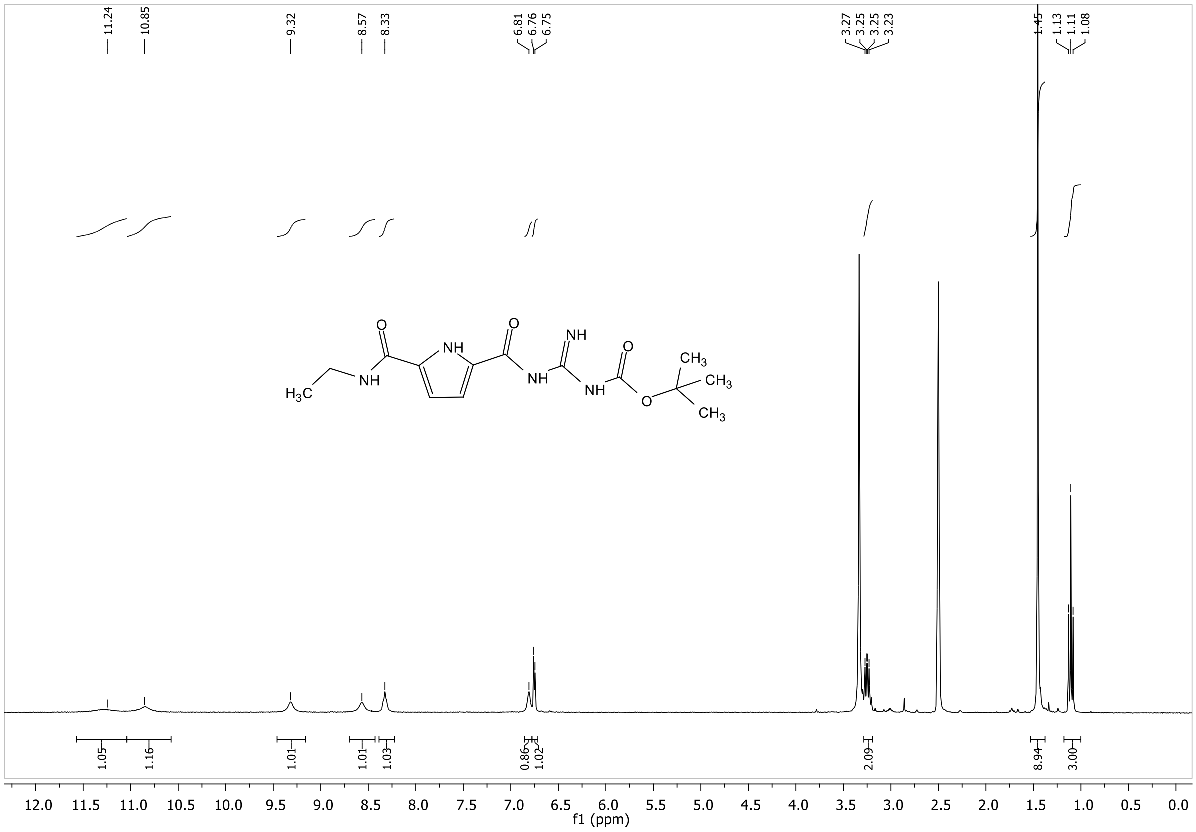

Figure S17: ^{1}H NMR spectrum of **L** (300 MHz, DMSO-d_6).

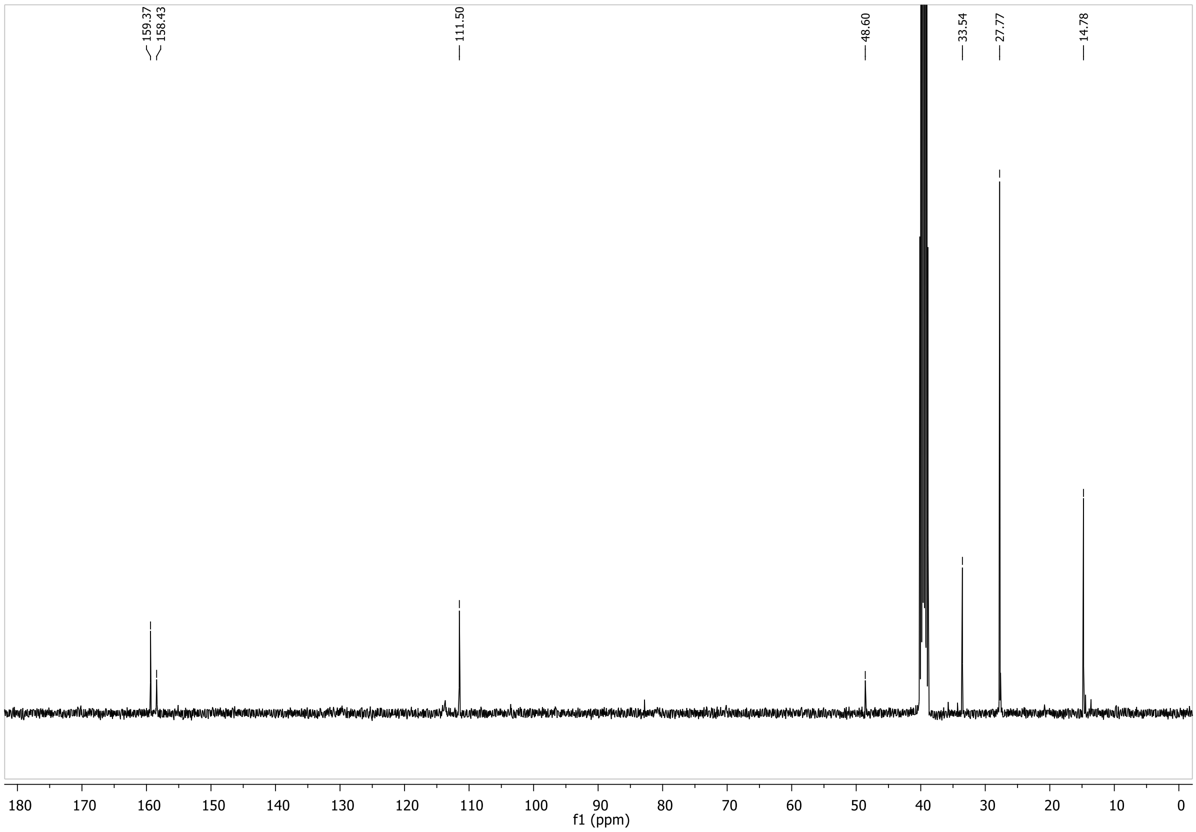

Figure S18: ^{13}C NMR spectrum of **L** (101 MHz, DMSO-d_6).

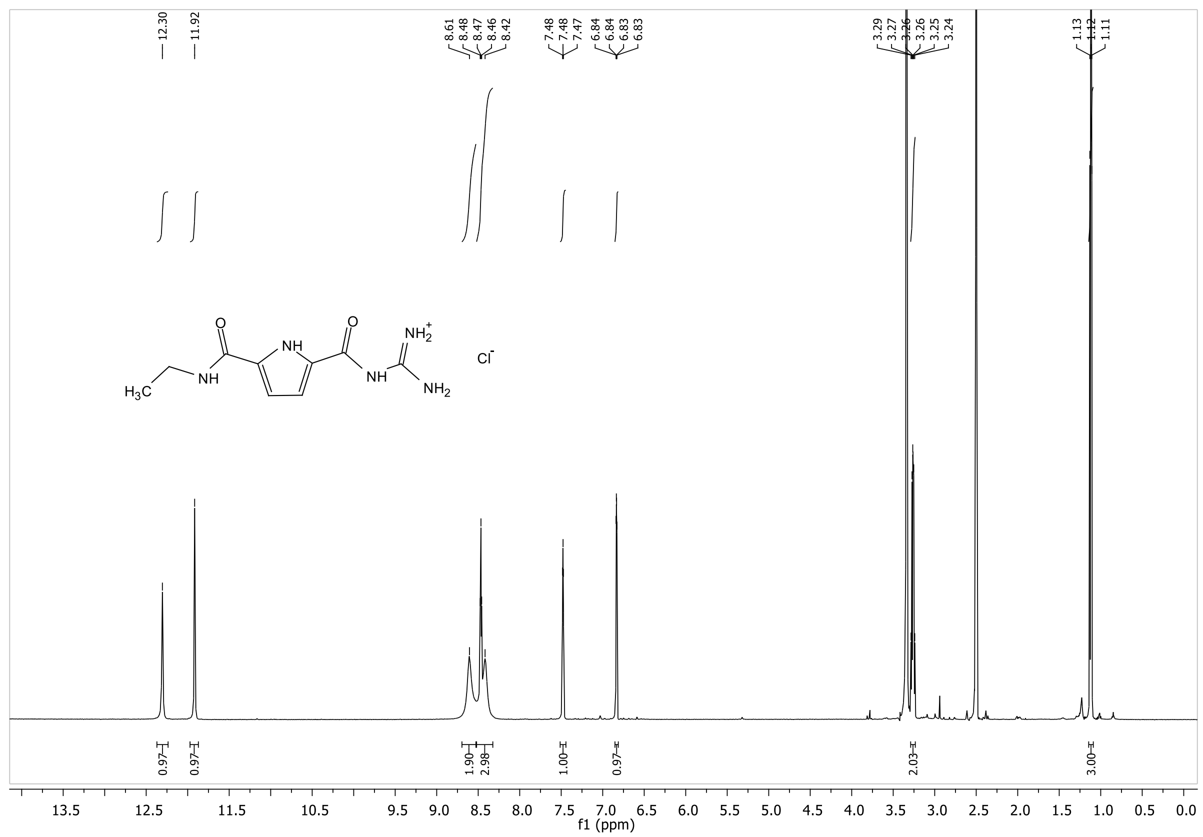

Figure S19: ^{1}H NMR spectrum of **1** (600 MHz, DMSO-*d*6).

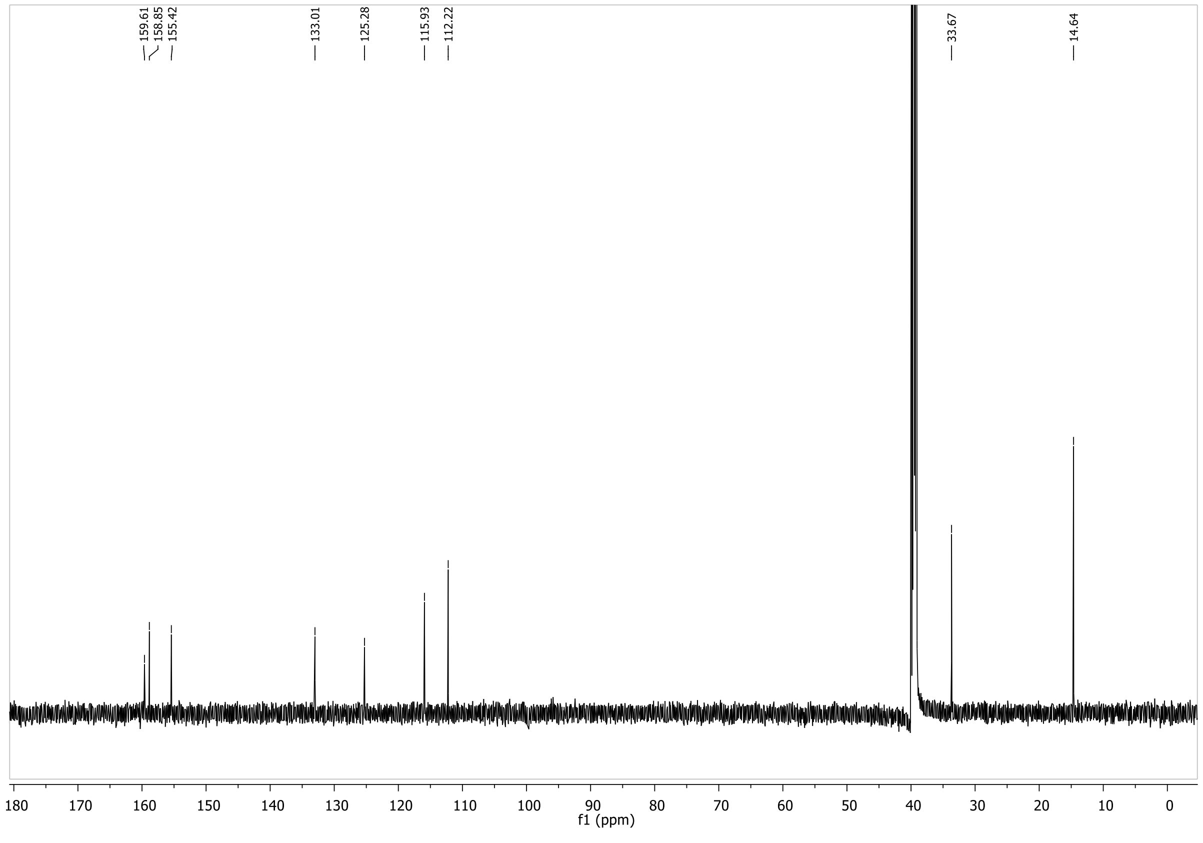

Figure S20: ^{13}C NMR spectrum of **1** (151 MHz, DMSO-*d*6).

Mass spectra

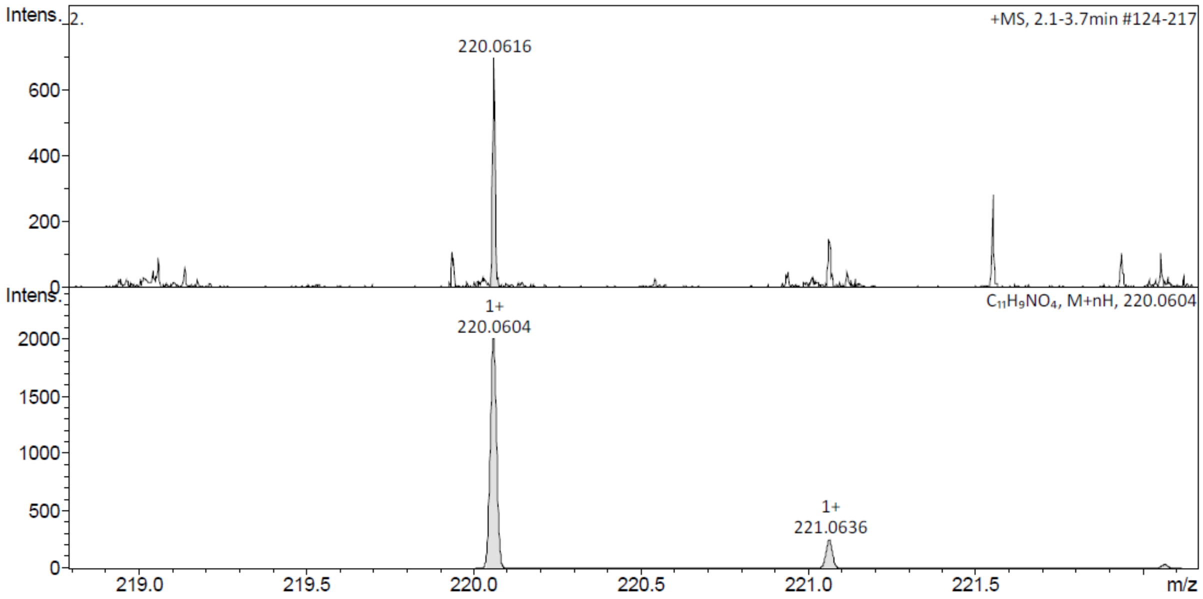

Figure S21: HR-ESI mass spectrum of **C** (positive ion mode, MeOH) and predicted mass spectrum of peaks which belongs to **C.**

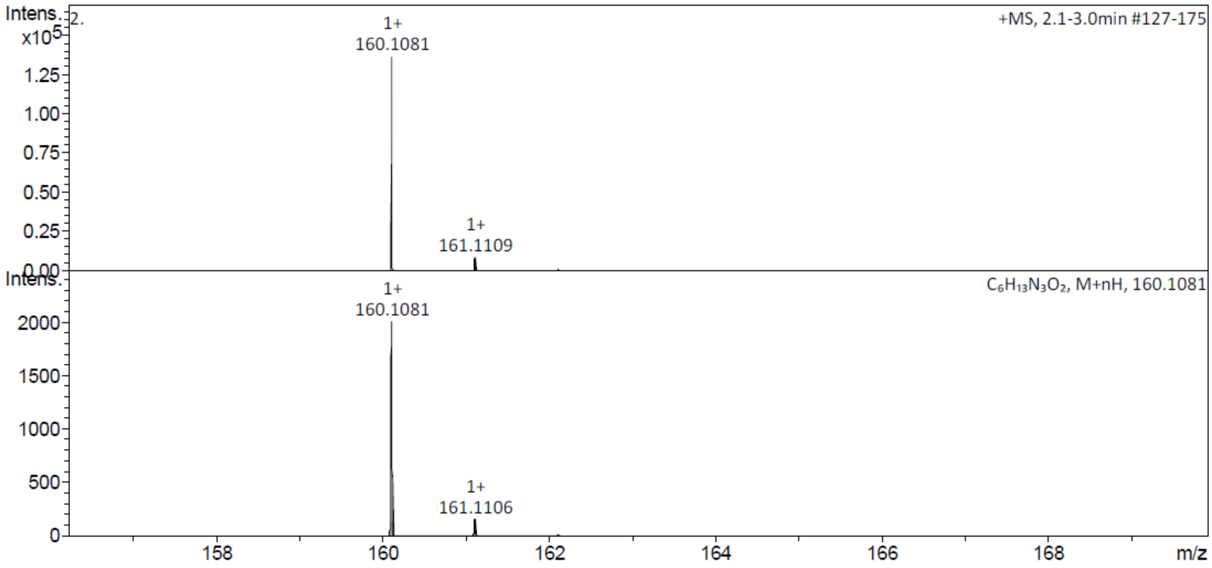

Figure S22: HR-ESI mass spectrum of **F** (positive ion mode, MeOH) and predicted mass spectrum of peaks which belongs to **F.**

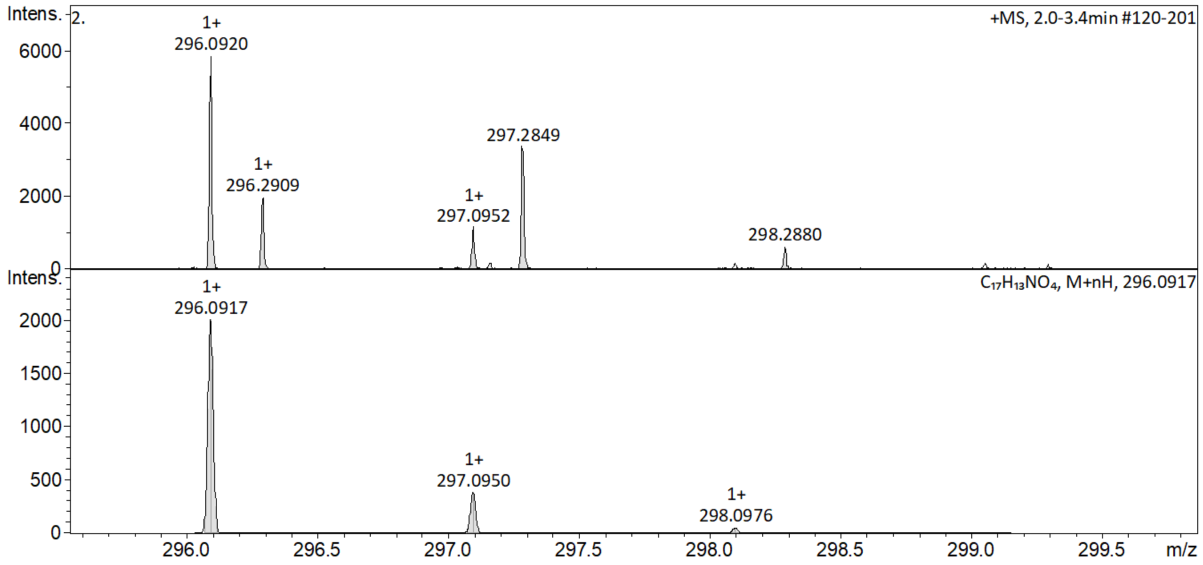

Figure S23: HR-ESI mass spectrum of **G** (positive ion mode, MeOH) and predicted mass spectrum of peaks which belongs to **G.**

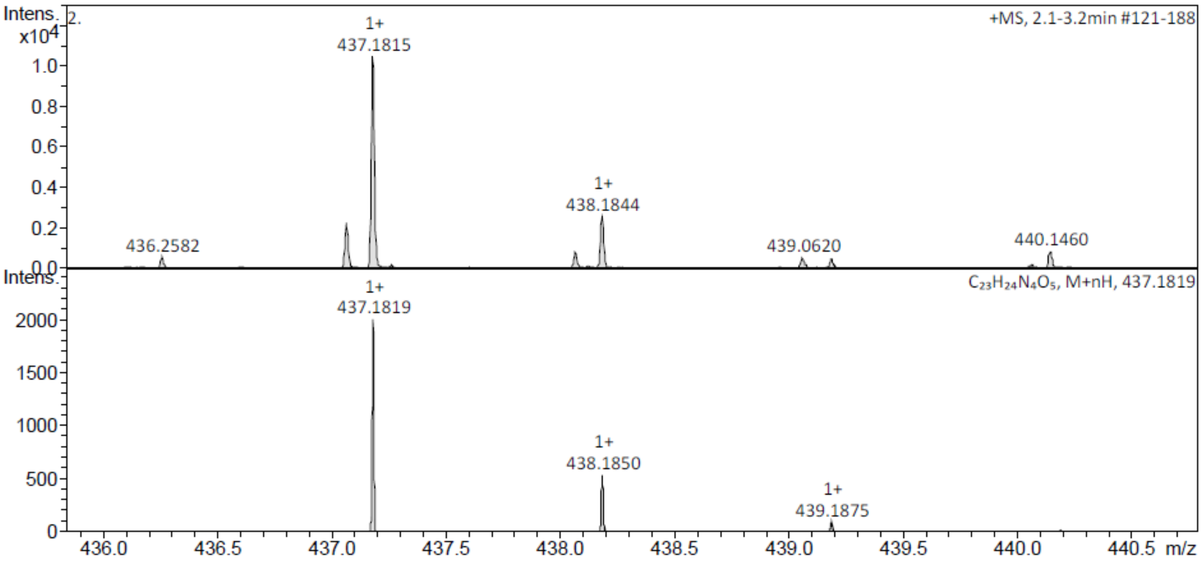

Figure S24: HR-ESI mass spectrum of **H** (positive ion mode, MeOH) and predicted mass spectrum of peaks which belongs to **H.**

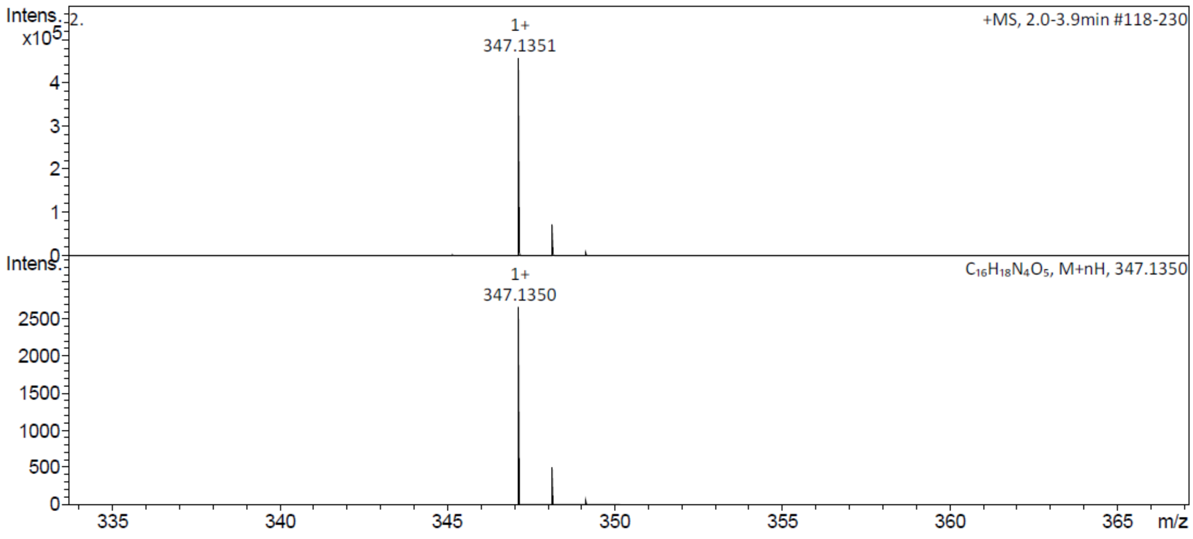

Figure S25: HR-ESI mass spectrum of **I** (positive ion mode, MeOH) and predicted mass spectrum of peaks which belongs to **I.**

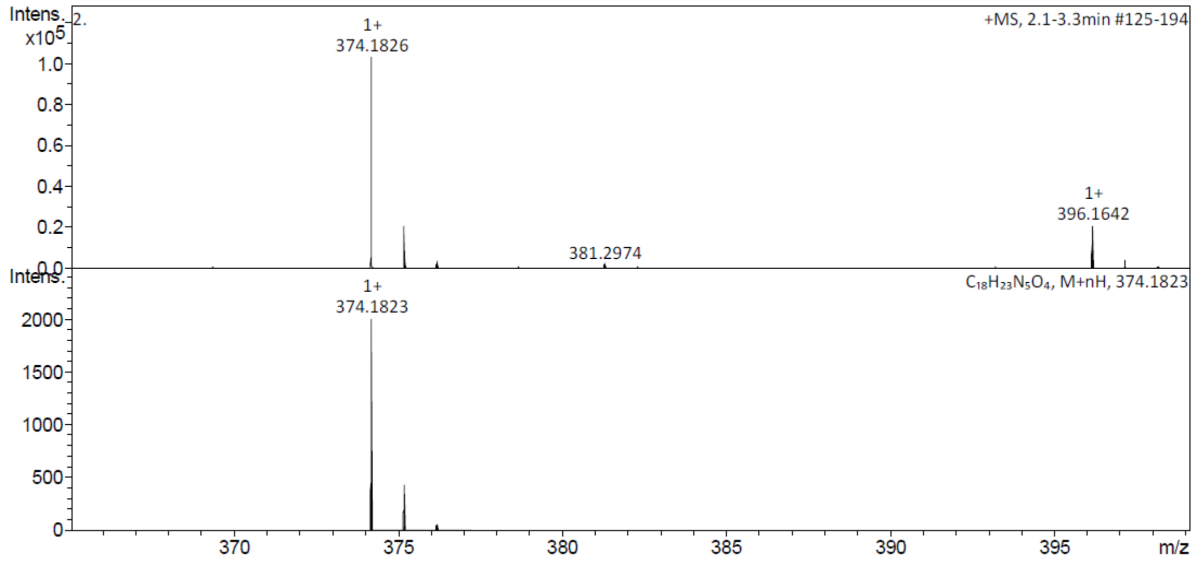

Figure S26: HR-ESI mass spectrum of **J** (positive ion mode, MeOH) and predicted mass spectrum of peaks which belongs to **J.**

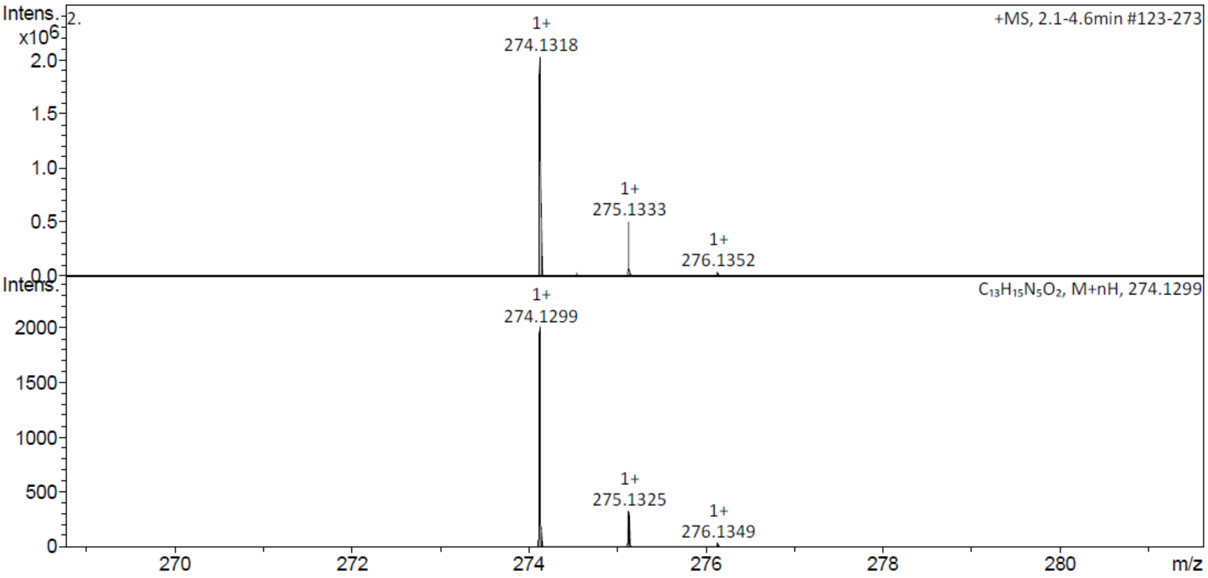

Figure S27: HR-ESI mass spectrum of **2** (positive ion mode, MeOH) and predicted mass spectrum of peaks which belongs to **2.**

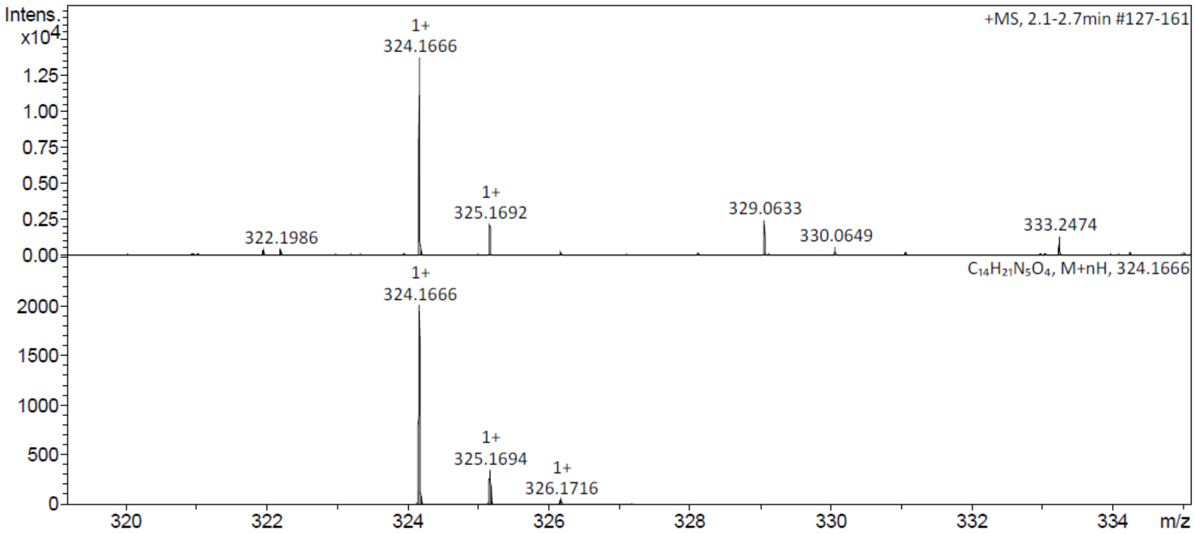

Figure S28: HR-ESI mass spectrum of **L** (positive ion mode, MeOH) and predicted mass spectrum of peaks which belongs to **L.**

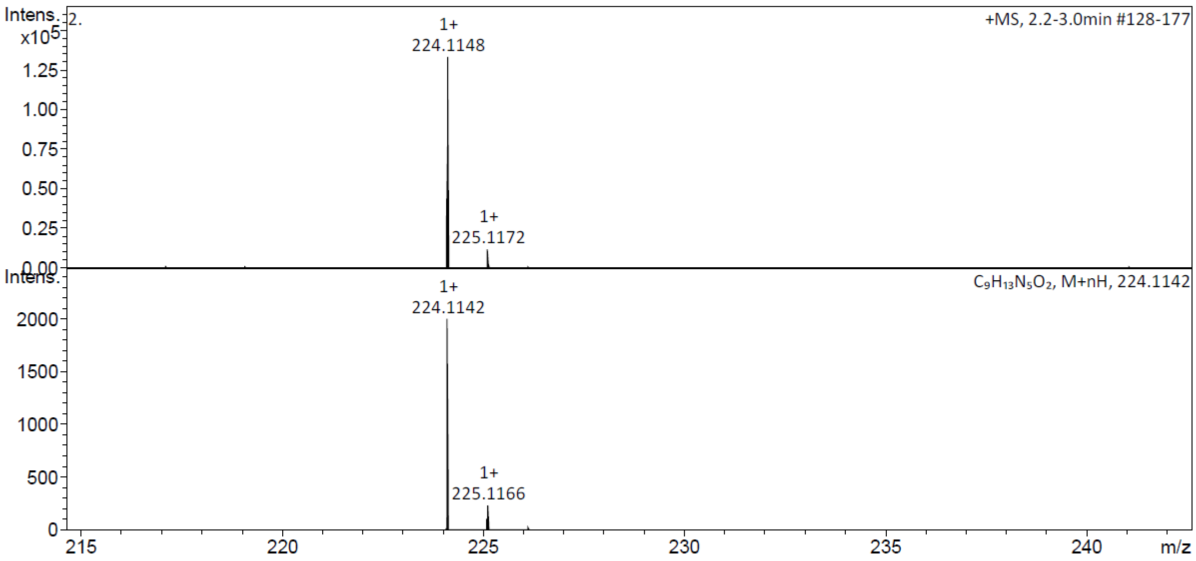

Figure S29: HR-ESI mass spectrum of **1** (positive ion mode, MeOH) and predicted mass spectrum of peaks which belongs to **1.**

Analytical HPLC

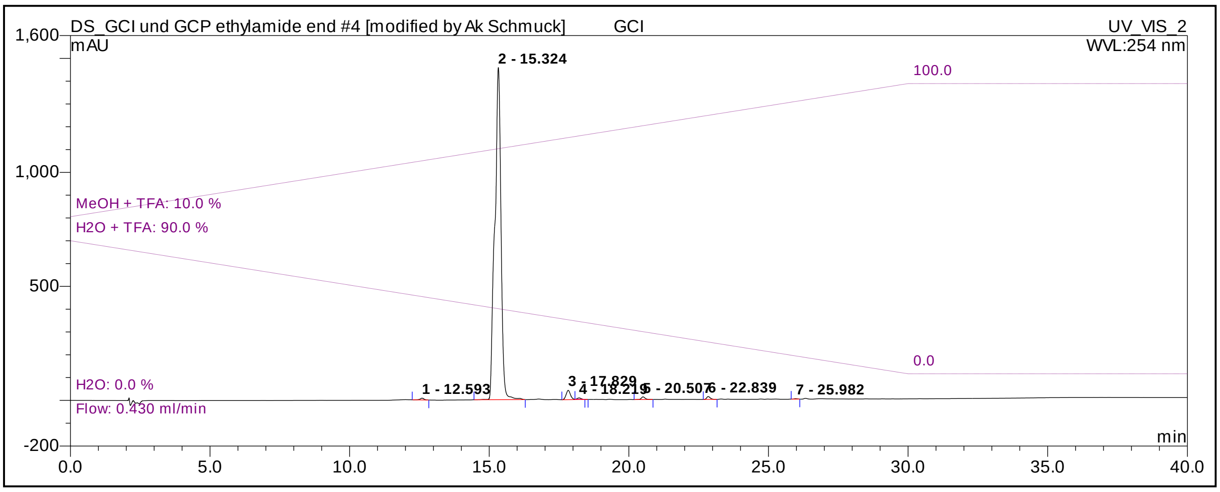

Figure S30: Analytical HPLC (RP 18 MeOH/H_2O + 0.1% TFA, 10% MeOH + 0.1% TFA to 100% MeOH + 0.1% TFA, gradient) **2**.

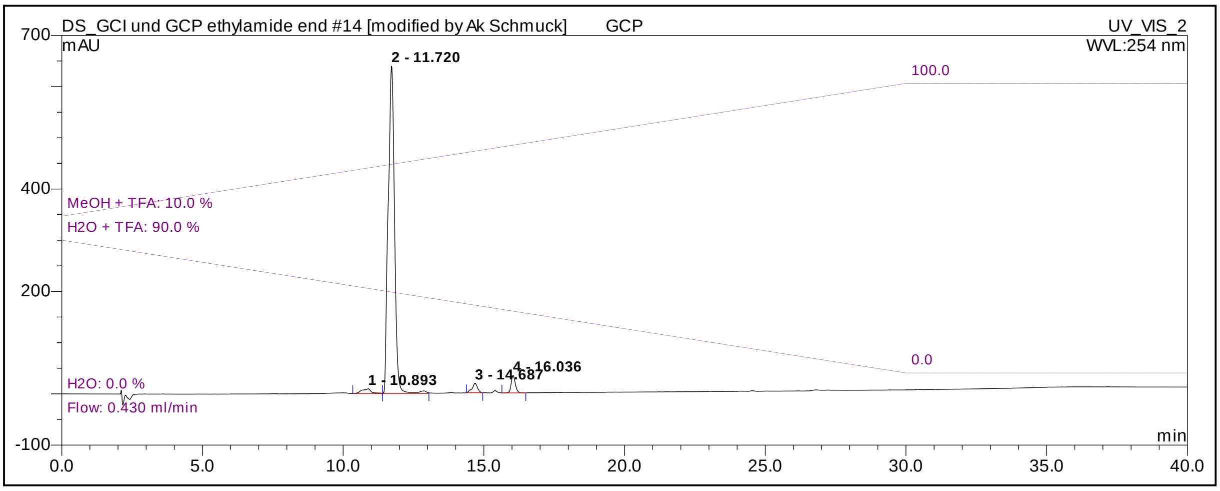

Figure S31: Analytical HPLC (RP 18 MeOH/H_2O + 0.1% TFA, 10% MeOH + 0.1% TFA to 100% MeOH + 0.1% TFA, gradient) **1**.

References

1. Frisch, M. J.; Trucks, G. W.; Schlegel, H. B.; Scuseria, G. E.; Robb, M. A.; Cheeseman, J. R.; Scalmani, G.; Barone, V. Petersson, G. A.; Nakatsuji, H.; et al., *Gaussian 16*, Rev. B01, Wallingford, CT, **2016**.
2. C. Rether and C. Schmuck, *Eur. J. Org. Chem.* **2011**, 1459–1466.
3. C. Schmuck *Chem. Commun.* **1999**, 843–844. doi:10.1039/A901126I
4. C. Schmuck, V. Bickert, M. Merschky, L. Geiger, D. Rupprecht, J. Dudaczek, P. Wich, T. Rehm and U. Machon *Eur. J. Org. Chem.* **2008**, 324–329.
